# Circulating Pneumolysin Is a Potent Inducer of Cardiac Injury during Pneumococcal Infection

**DOI:** 10.1371/journal.ppat.1004836

**Published:** 2015-05-14

**Authors:** Yasir Alhamdi, Daniel R. Neill, Simon T. Abrams, Hesham A. Malak, Reham Yahya, Richard Barrett-Jolley, Guozheng Wang, Aras Kadioglu, Cheng-Hock Toh

**Affiliations:** 1 Department of Clinical Infection, Microbiology and Immunology, Institute of Infection and Global Health, University of Liverpool, Liverpool, United Kingdom; 2 Department of Musculoskeletal Biology, Institute of Aging and Chronic Diseases, University of Liverpool, Liverpool, United Kingdom; 3 Roald Dahl Haemostasis & Thrombosis Centre, Royal Liverpool University Hospital, Liverpool, United Kingdom; Boston Children’s Hospital, UNITED STATES

## Abstract

*Streptococcus pneumoniae* accounts for more deaths worldwide than any other single pathogen through diverse disease manifestations including pneumonia, sepsis and meningitis. Life-threatening acute cardiac complications are more common in pneumococcal infection compared to other bacterial infections. Distinctively, these arise despite effective antibiotic therapy. Here, we describe a novel mechanism of myocardial injury, which is triggered and sustained by circulating pneumolysin (PLY). Using a mouse model of invasive pneumococcal disease (IPD), we demonstrate that wild type PLY-expressing pneumococci but not PLY-deficient mutants induced elevation of circulating cardiac troponins (cTns), well-recognized biomarkers of cardiac injury. Furthermore, elevated cTn levels linearly correlated with pneumococcal blood counts (*r*=0.688, *p*=0.001) and levels were significantly higher in non-surviving than in surviving mice. These cTn levels were significantly reduced by administration of PLY-sequestering liposomes. Intravenous injection of purified PLY, but not a non-pore forming mutant (PdB), induced substantial increase in cardiac troponins to suggest that the pore-forming activity of circulating PLY is essential for myocardial injury in vivo. Purified PLY and PLY-expressing pneumococci also caused myocardial inflammatory changes but apoptosis was not detected. Exposure of cultured cardiomyocytes to PLY-expressing pneumococci caused dose-dependent cardiomyocyte contractile dysfunction and death, which was exacerbated by further PLY release following antibiotic treatment. We found that high PLY doses induced extensive cardiomyocyte lysis, but more interestingly, *sub-lytic* PLY concentrations triggered profound calcium influx and overload with subsequent membrane depolarization and progressive reduction in intracellular calcium transient amplitude, a key determinant of contractile force. This was coupled to activation of signalling pathways commonly associated with cardiac dysfunction in clinical and experimental sepsis and ultimately resulted in depressed cardiomyocyte contractile performance along with rhythm disturbance. Our study proposes a detailed molecular mechanism of pneumococcal toxin-induced cardiac injury and highlights the major translational potential of targeting circulating PLY to protect against cardiac complications during pneumococcal infections.

## Author Summary

Cardiac complications frequently accompany invasive disease caused by the pathogen *Streptococcus pneumoniae* and are associated with significant increases in mortality, however the underlying mechanisms remain elusive. Here, we describe a new mechanism by which pneumococci in the blood stream induce elevation of circulating cardiac troponins; proteins that are markers of cardiac injury and cause inflammatory cell infiltration into the myocardium. We demonstrate that this process is mediated by the circulating pneumococcal toxin pneumolysin (PLY). We also show that antibiotic treatment can exacerbate cardiac injury and dysfunction following pneumococcal infection due to bacterial lysis and the release of PLY, which represents a further mechanistic explanation for the process of cardiac scarring and inflammation. We demonstrate that in addition to its role in inducing death of cardiac cells at high concentrations, PLY at lower (non-lytic) concentrations trigger a range of cellular events starting with cellular membrane pore-formation, substantial calcium overload which then mediates mechanical and electrical disturbance to the function of cardiac cells. Our work proposes that novel translational strategies to detect and neutralize circulating PLY during pneumococcal disease could be utilized to assess disease severity and should be targeted for prevention/treatment purposes.

## Introduction

*Streptococcus pneumoniae* (the pneumococcus) is a major human pathogen responsible for severe invasive diseases such as pneumonia, sepsis and meningitis in young children and immunocompromised individuals worldwide [1], while also being the main cause of community acquired pneumonia (CAP) in the elderly in the UK and USA. There is now growing awareness of associated cardiac events (ACEs) during pneumococcal infection, particularly in the context of CAP in which over a quarter of the patients develop ACEs. These include arrhythmia, cardiac failure and acute coronary syndrome [2–5], which are associated with an increased overall risk of mortality from 13.9% to 36% [4–6]. In addition to these observations, elevated levels of cardiac troponins, which are well-recognized biomarkers of cardiac injury have been shown to adversely affect prognosis in pneumococcal pneumonia [7]. Pneumococcal infection is also a common cause of severe sepsis and septic shock [8,9]. Cardiac depression occurs in 40–50% of patients with severe sepsis and raises the mortality rate to 80–90% [10,11]. Cardiomyocyte damage marked by elevated troponin levels occurs in ~61% [12] of patients with severe sepsis. However, the underlying mechanisms of these cardiac complications remain poorly understood.

Increased myocardial oxygen demand, lowered myocardial oxygenation, vascular endothelial dysfunction and the effects of increased inflammatory cytokine levels [2,13,14] have been proposed as mechanisms contributing to the development of ACEs and cardiac injury in pneumococcal infection. However, these mechanisms are not pathogen-specific. A more direct role is suggested by the finding that pneumococcal pneumonia is much more likely to be associated with acute cardiac complications compared to other pathogens [14]. Importantly, pneumococcal pneumonia is also a strong independent risk factor for the development of cardiac complications, including arrhythmia [2]. In addition, the risk of ACEs and associated mortality remains high in the first few days of pneumococcal infection despite appropriate antibiotic treatment [15]. Recently, a study by Brown *et al* provided new evidence that pneumococci could translocate across the myocardial vasculature to cause microlesions within the myocardial tissue that disrupt cardiac function [16].

*Streptococcus pneumoniae* produces a range of virulence factors that promote bacterial pathogenesis [17]. One such virulence factor is the cholesterol-binding haemolytic cytotoxin pneumolysin (PLY), which is a 53 KDa protein that is closely associated with the development of invasive disease and inflammation [18] and released by pneumococci upon cell lysis. The cytotoxicity of PLY is attributed to its potent pore-forming ability on cholesterol containing cell membranes and subsequent disruption of intracellular Ca^2+^ homeostasis [19,20] although it also has a wide range of effects on cell signalling at sub-lytic concentrations, including induction of apoptosis [21] and activation of the NLRP3 inflammasome [22]. Ca^2+^ dynamics are considered central to cardiac contractile rhythm and force [23]. As part of the normal physiological adaptation to increased metabolic demands, an increased release of catecholamines takes place to increase both force and rate of cardiac contraction by increasing Ca^2+^ entry into cardiomyocytes through the inward Ca^2+^ current *I*_Ca_ [24]. This increment in intracellular Ca^2+^ concentration [Ca^2+^]_i_ is physiological but, beyond certain limits, pathological Ca^2+^ overload develops to result in cellular injury as well as electrical and mechanical dysfunction [25]. As such, the observation that PLY can mediate pathological Ca^2+^ influx into various mammalian cells may be particularly relevant to cardiomyocyte dysfunction during invasive pneumococcal disease (IPD). Furthermore, Ca^2+^ also regulates pivotal signalling processes in cardiomyocytes including post-translational modification of myofilament proteins and endoplasmic reticulum (ER) stress [26,27]. In particular, cardiac troponin (cTn) phosphorylation by protein kinase C (PKC) has direct functional implications on contractile performance [27] and has been widely studied. This leads us to hypothesize that circulating PLY is the key virulence factor to induce cardiomyocyte dysfunction by affecting intracellular Ca^2+^ homeostasis and relevant signalling pathways.

In this study, we demonstrate that PLY plays a key role in pneumococcal-induced cardiomyocyte injury and in elevation of circulating cardiac troponins in a mouse model of IPD. The underlying mechanisms are attributed to the pore-forming properties of circulating PLY, which directly cause greatly increased Ca^2+^ influx into cardiomyocytes and subsequent Ca^2+^ overload. The profound increase in diastolic [Ca^2+^]_i_ attenuates the intracellular Ca^2+^ transient amplitude and enhances activation of Ca^2+^-sensitive pathways that are known to be associated with myocardial dysfunction, such as PKCα-cTnI axis and ER stress to reduce cardiomyocyte contractile performance and also lead to cell injury. Furthermore, this study provides novel therapeutic evidence that neutralization of circulating PLY in mice using specially engineered PLY-sequestering liposomes [28] can significantly attenuate cardiac injury during IPD.

## Results

### Only PLY-expressing strains induce cardiomyocyte injury *in vivo*

**Fig 1:**
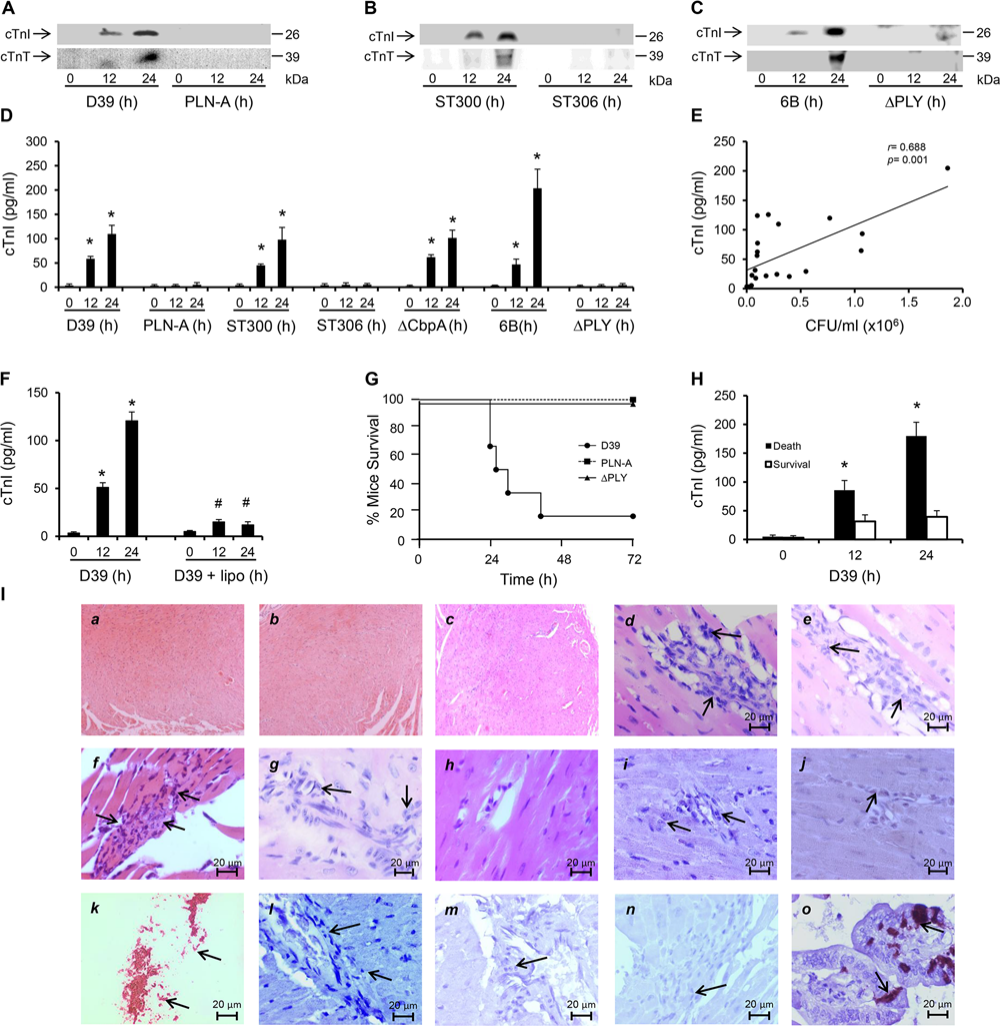
PLY-expressing but not PLY-deficient pneumococci induce cardiac injury and inflammation. (**A-C**) Representative Western blots showing circulating cTnI and cTnT in murine plasma following i.v. injection of D39/PLN-A (n = 4) (1x10^6^ CFU) (A) serotype-1, sequence type 300 and 306 (n = 5) (1x10^6^ CFU) (B), and serotype 6B and PLY-deletion pneumococci (ΔPLY) (1x10^6^ CFU) (C). (**D**) Mean±SD of circulating cTnI from 4 mice (each group) that survived at 24 h after treatment with D39, PLN-A, ST300, ST306, ΔCbpA, 6B and ΔPLY (1x10^6^ CFU each). *p<0.05 as compared to 0 h. (**E**) Linear correlation between pneumococcal CFU counts and circulating cTnI levels at various time points (n = 21). (**F**) Circulating levels of cTnI in plasma of mice following i.v. injection of D39 (1x10^6^ CFU) with or without liposomes (lipo). (n = 3 each group). *p<0.05 as compared to 0 h, #*p*<0.05 as compared to D39 group. (**G**) Survival curves of mice infected (i.v.) with D39,PLN-A and ΔPLY (n = 10 for D39, n = 5 for PLN-A, n = 4 for ΔPLY). (**H**) Circulating levels of cTnI in plasma of mice that died and those that survived within the first 30 h post-D39 infection (n = 4 each group). *p<0.05 as compared to survival group. (**I**) Pathological examination of murine heart sections after infection with D39, serotype-1, serotype 6B and ΔCbpA pneumococci. H&E representative images (*a-h*) of murine heart sections under x4 magnification (*a-c*) and x60 magnification (*d-h*). Hearts from mice infected with D39 (30 h post infection, *d*), serotype-1 *(e)*, 6B *(f*) and ΔCbpA (*g*) show inflammatory cell infiltration (arrows) at x60 magnification, these are absent in a normal heart section (*h*). (*i-j*) Immunohistochemistry images showing absence of pneumococcal capsule staining in heart from mice infected with D39 (*i*) and serotype-1 (*j*), despite presence of inflammatory cell infiltrations (arrows). (*k*) Fresh pneumococci (D39) (arrows) were stained in parallel, as a positive control for pneumococcal capsule staining. (*l-n*) Representative immunohistochemistry images showing absence of active caspase-3 staining in heart section from mice infected with D39 (*l*), serotype-1 (*m*) and 6B (*n*) despite presence of inflammatory cell infiltrations (arrows). (*o*) Gut microvilli of a septic mouse showing positive active caspase-3 signal (arrows), was used as a positive control for active caspase-3 staining.

To examine the effects of invasive pneumococcal infection on cardiac injury *in vivo*, wild type (WT) *S*. *pneumoniae* serotype 2 (strain D39) or its pneumolysin deficient isogenic strain (PLN-A [18,29]) (1x10^6^ CFU in 50 μl saline) were administered intravenously (i.v.) into mice. We found that cardiomyocyte injury biomarkers, i.e. cardiac troponin I (cTnI) and T (cTnT), were detectable in the circulation of mice infected with D39 as early as 12 h with much higher levels 24 h post-infection (Fig 1A). However, neither cTnI nor cTnT were detected in plasma of PLN-A-infected mice (Fig 1A) suggesting that PLY is key to inducing cardiomyocyte injury during IPD. D39 and PLN-A blood counts were comparable at 12 hours post-infection (S1A Fig), suggesting that the presence/absence of cardiac troponin release was not due to a higher bacterial burden in blood with D39 but instead due to the presence/absence of PLY in the two strains. To confirm this, we performed similar infections with clinical invasive disease isolates of serotype-1 pneumococci of a sequence type producing haemolytic PLY (ST300) and with a sequence type that produces a non-haemolytic PLY (ST306), and found that both cTnI and cTnT are elevated with ST300 but not with ST306 (Fig 1B). This result suggests that only active PLY-expressing pneumococci are able to induce cardiac injury. Furthermore, infecting mice (i.v 10^6^ CFUs) with a clinical invasive disease isolate of serotype 6B produced similar release of cardiac troponins into the circulation (Fig 1C), whereas an infection with a complete PLY-deletion mutant (ΔPLY) [30] did not yield cardiac troponin release (Fig 1C).

In a recent publication using a model of peritoneal infection, Brown *et al* reported that myocardial microlesion development was largely dependent on pneumococcal translocation to the myocardial tissue, which required the pneumococcal adhesin Choline binding protein A (CbpA) and that use of a CbpA-deficient pneumococcus on the serotype 4 background (TIGR4) resulted in significant reduction in microlesion formation within 24 h of infection [16]. However, using a CbpA-deficient pneumococcus (ΔCbpA) on the serotype 2 background (D39) in a model of pneumococcal sepsis, we found that circulating cardiac troponins (I and T) were significantly elevated as early as 12 h post infection, with higher levels at 24 h (Figs 1D and S2A). Levels of cTnI in the circulation of mice infected with D39, serotype-1 (ST300), serotype 6B and CbpA-deficient pneumococci were comparable and reached levels as high as ~110–200 pg/ml (Fig 1D), approximately 10-fold higher than the normal levels observed in humans (~14 pg/ml). These results suggest that the pneumococcal adhesin CbpA has no major role in inducing cardiac injury in our model of IPD and that cardiac troponins are only elevated in the circulation of mice challenged with PLY-expressing pneumococci. Furthermore, we found a significant linear correlation (*r* = 0.688, *p* = 0.001) between pneumococcal burden in the blood and circulating cTnI levels (Fig 1E), suggesting an association between the severity of bacteraemia and that of cardiac injury. In recent work, we have shown that circulating PLY as a major source of toxicity during IPD can be directly targeted by specifically engineered liposomes that compete with host cells for PLY-binding and result in significant improvement in mice survival [28]. We show here that injecting mice i.v. with toxin-sequestering liposomes (100 mg/kg) 30 min after injecting them with WT D39 pneumococci, resulted in a significant reduction in circulating cTnI by 12 and 24 h post-infection to levels as low as 12–15 pg/ml (Figs 1F and S1B). These results strongly indicate that circulating PLY is the major source of cardiac injury. Seventy percent of D39-challenged mice died within 30 h of infection, whereas none of the PLY-deficient (PLN-A or ΔPLY)-infected mice (0/5) died (Fig 1G), in line with results from previous studies [30]. Interestingly, mice that died within 30 h of D39 infection, had significantly higher circulating cTnI levels at 12 h and 24 h (179±23 pg/ml) than mice that survived (39±10 pg/ml) (Fig 1H) suggesting that elevated cardiac troponins in this context could be reflective of greater disease severity and worse outcomes. Collectively, these data suggest that PLY-induced cardiac injury potentially contribute to increased mortality during invasive pneumococcal infection.

Histo-pathological examination of heart sections from mice infected with either serotype 2 (D39) or a highly invasive clinical serotype-1 isolate and serotype 6B, as well as PLY-expressing but CbpA-deficient pneumococci showed no evidence of gross myocardial abnormalities (Figs 1I, *a-c* and S2B) but revealed inflammatory cell infiltration into the myocardium (Fig 1I, *d-g*). Furthermore, we were unable to identify pneumococcal colonization in any of the heart sections (Figs 1I, *i-j* and S2B). Careful histo-pathological examination of cardiac tissue sections from mice that died between 24 h and 96 h after infection (with D39, serotype-1, 6B and CbpA-deficient pneumococci) showed only minimal numbers of inflammatory cell infiltration in loci around microvessels within the myocardium. We also investigated whether apoptotic changes were affecting the myocardium, but were unable to detect activation of apoptotic markers in murine hearts infected with serotype-2, serotype-1 or 6B even after 96 h of infection (Fig 1I, *l-n*). Collectively, our data strongly suggest that circulating PLY, released by pneumococci in the bloodstream, mediates myocardial inflammatory changes and injury but these are not accompanied by pneumococcal colonization or apoptotic changes of the myocardium in our model of IPD.

### Circulating pore-performing PLY mediates myocardial injury and death

**Fig 2:**
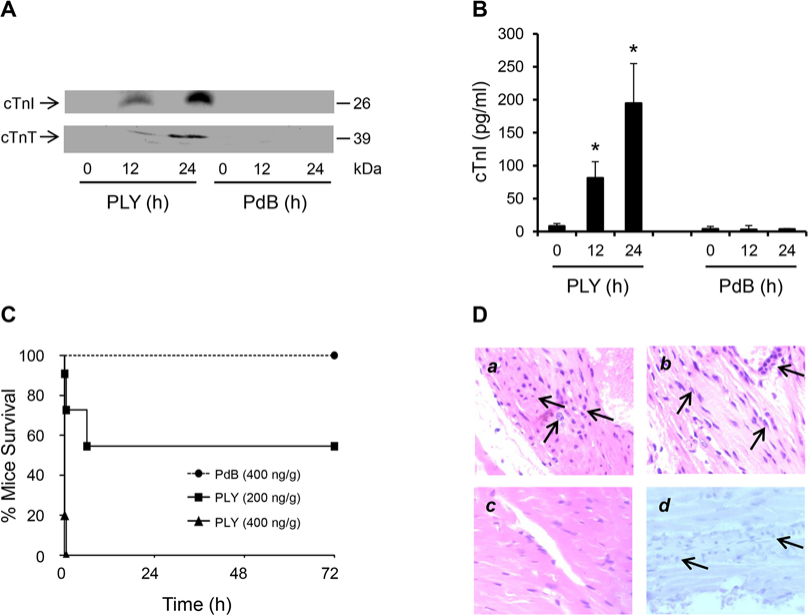
Circulating pore-forming PLY mediates cardiac injury and inflammation *in vivo*. (**A**) Representative Western blots showing circulating cTnI and cTnT in murine plasma following i.v. injection of PLY (200 ng/g) and PdB (400 ng/g). (**B**) Mean±SD of circulating cTnI from 4 mice (each group) that survived at 24 h after treatment with PLY and PdB. *p<0.05 as compared to PdB group. (**C**) Survival curves of mice injected (i.v.) with PLY (400 and 200 ng/g) or PdB (400 ng/g) (n = 8 each group). (**D**) Pathological examination of murine heart sections after infection with purified PLY (200 ng/g) (*a*, *b* and *d*) and PdB (400 ng/g) (*c*). H&E representative images (*a-c*) of murine heart sections under x60 magnification showing inflammatory cell infiltrations (arrows) in mice dying within 6 hours (*a*) and those surviving for 24 hours (*b*). (*c*) PdB injection causes no pathological abnormality in murine myocardium. (*d*) Absence of active caspase-3 staining in murine myocardium following PLY injection.

To test if circulating PLY could cause cardiac injury directly, purified PLY was injected through mouse tail veins with its non-pore forming mutant PdB as a control. We found that injection of PLY (200 ng PLY/g mouse) induced high levels of circulating cTnI and cTnT, 81±6 pg/ml at 12 h and 195±40 pg/ml at 24 h after injection (Fig 2A and 2B). In contrast, PdB (400 ng/g) did not produce detectable levels of circulating cardiac troponins in mice (Fig 2A and 2B), indicating that the cellular pore-forming properties of PLY are central to cardiac injury. PLY at a concentration of 400 ng/g caused death in all treated mice within 30 min (Fig 2C). PLY at 200 ng/g induced a bimodal response causing 5/11 mice to die within the first 6 h and the remainder (6/11) survived until euthanized at 72 h (Fig 2C). Whilst cardiac injury may contribute to the early deterioration seen in approximately half of mice treated with lower dose PLY, it is unlikely to be the only factor and extensive cytolysis is also likely to play a role. We found no evidence of elevated circulating cardiac troponins in mice dying within 6 h post injection of PLY (200 ng/g), but this is unsurprising given that cardiac troponins typically peak in the circulation 12–24 h after cardiac injury [31,32]. Nevertheless, histo-pathological examination of the myocardium did reveal signs of inflammatory cell infiltration as early as 6 h post injection of PLY (Fig 2D *a*) but these were also observed in mice that survived for up to 24 h (Fig 2D *b*) and, as expected, were absent when PdB was injected (Fig 2D *c*). We found no activation of apoptosis in the myocardium following PLY injection (Fig 2D *d*), confirming our findings with PLY-expressing pneumococci (for serotypes 1, 2 and 6B) (Fig 1I).

These data further confirm that circulating PLY is a critical pathological factor and that cardiac injury and inflammation contribute to pathology.

### Antibiotics lyse pneumococci to release PLY and exacerbate cardiomyocyte injury

**Fig 3:**
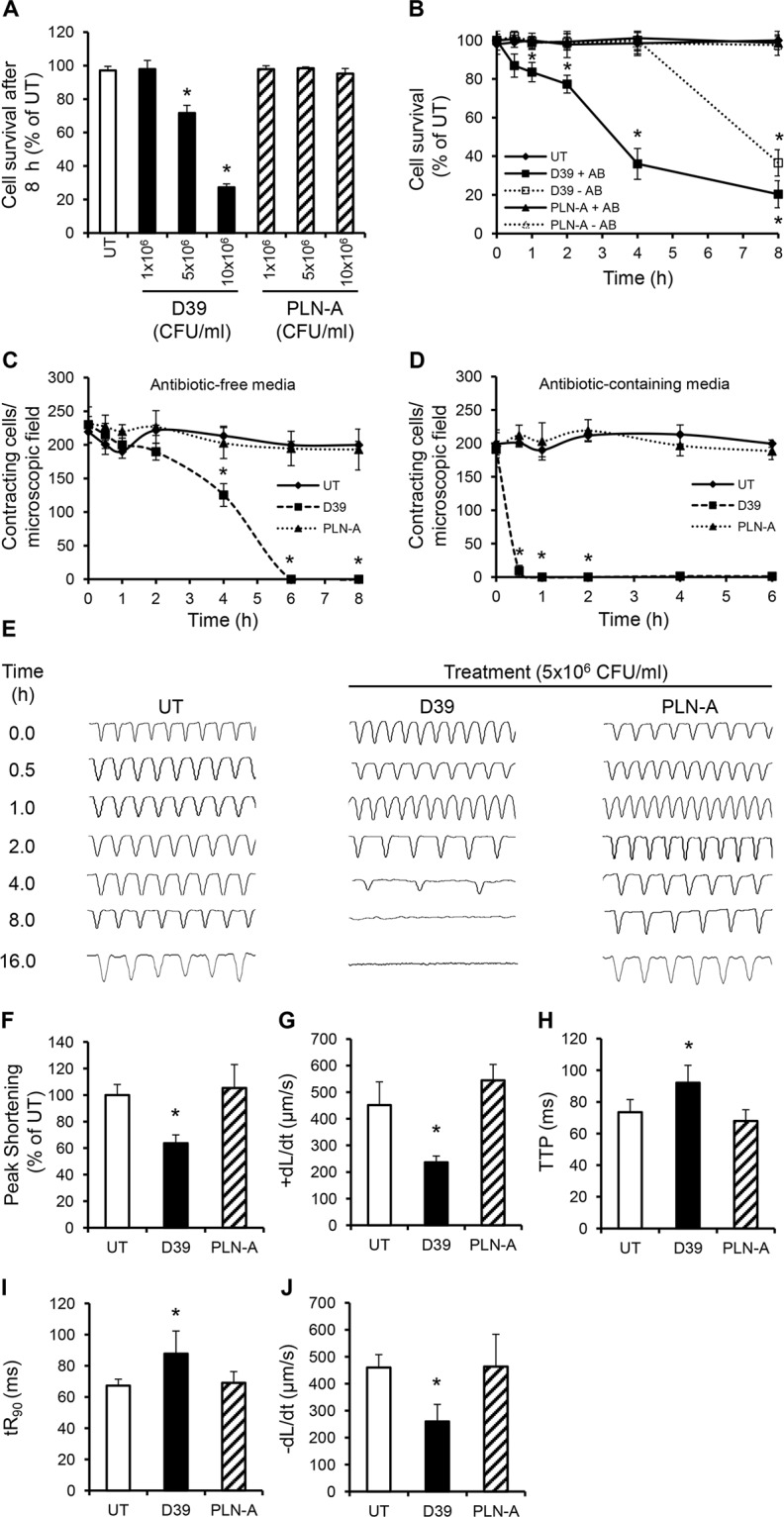
Wild type (D39) but not PLY-deficient (PLN-A) pneumococci cause cardiomyocyte dysfunction and death *in vitro*. (**A**) Viability of HL-1 cells was assessed at 8 h after incubation with increasing CFUs of D39 and PLN-A. Viability of untreated cells (UT) was set at 100%. Data are presented as Mean±SD (n = 4). (**B**) Time course of HL-1 cell viability after incubation with 5x10^6^ CFU/ml D39 or PLN-A in the presence or absence of antibiotics (AB). Data are presented as Mean±SD (n = 4). (**C**) and (**D**) Effects of D39 and PLN-A (5x10^6^ CFU/ml) on the total number of spontaneously contracting HL-1 cells over time using antibiotic-free (C) and antibiotic-containing media (D). Data are presented as Mean±SD (n = 4). (**E**) Effects of D39 and PLN-A (5x10^6^ CFU/ml) on the rhythm and rate of contraction of HL-1 cells over time. Typical traces of spontaneous contraction are presented. (**F-J**) Effects of D39 and PLN-A (5x10^6^ CFU/ml) on Peak Shortening (F), maximum velocity of shortening (+dL/dt) (G), Time to Peak (TTP) (H), Time to 90%-re-lengthening tR_90_ (I) and maximum velocity of relaxation (-dL/dt) (J) of HL-1 cells after 4 h treatment are presented as Mean±SD (n = 9 from 3 independent experiments). *ANOVA test, *p*<0.05.

To examine whether and how cardiomyocytes may be affected by pneumococcal infection, HL-1 cells, which have typical phenotypic features of adult cardiomyocytes [33,34] were infected with either wild type WT D39 or PLY-deficient pneumococci (PLN-A). Exposure to WT D39 resulted in a significant, dose-dependent reduction in cell viability (Fig 3A); dropping to 71.7±4.5% at 5x10^6^ colony forming units (CFU)/ml and 27.2±2.1% at 10^7^ CFU/ml by 8 h post-infection (*p*<0.05). No reduction in cardiomyocyte viability was observed when cells were infected with PLY-deficient PLN-A, even at the highest CFU dose of 10^7^ CFU/ml. Interestingly, we found that cell viability declined much earlier when WT D39 pneumococci were lysed in the presence of antibiotics (from 1 h) than in its absence (after 4 h) (Fig 3B). No reduction in viability was seen when PLN-A pneumococci were lysed with antibiotics, clearly suggesting that released PLY was responsible for the effect seen. This is in line with the known lytic release properties of PLY.

A significant decrease in the total number of spontaneously contracting cardiomyocytes was observed following WT D39 infection, with 230±26 cells/microscopic field before treatment reducing to 180±12 cells/field after 2 h of incubation with 5x10^6^ CFU/ml D39 (Fig 3C). This value continued to decline with time; 125±16 cells/field at 4 h and no contracting cells were observed at 6 h (Fig 3C). When cardiomyocytes were incubated with D39 in media containing antibiotics, the reduction in the number of spontaneously contracting cells was even more dramatic with ~10±8 cells/ field within 30 min and no contracting cells after 60 min (Fig 3D). Once again, no such reductions were observed when cardiomyocytes were infected with PLN-A, suggesting that PLY release following D39 lysis was able to severely affect cardiomyocyte function.

Using a cell edge-recognition system (IonOptix) to record real-time changes in cardiomyocyte shortening (i.e. contractility) [35], infection with D39 at 5x10^6^ CFU/ml induced slow and irregular beats in a time-dependent manner (Fig 3E) suggesting major rhythm disturbance. After 4 h of infection with D39, Peak Shortening (indicator of peak contraction) dropped to 63.6±6.3% (Fig 3F, *p* = 0.015) and the maximum velocity of shortening (+dL/dt) (indicator of maximum velocity of ventricular muscle contraction) fell from 423±25 μm/s (untreated control) to 236±23 μm/sat 4 h (Fig 3G, *p* = 0.003). Other parameters of contractility, such as time to peak “TTP”, time to 90% re-lengthening “tR_90_” (indicators of systolic and diastolic durations, respectively) and maximum velocity of re-lengthening “–dL/dt” (indicator of maximum velocity of ventricular muscle relaxation) were also negatively affected after 4 h of incubation with D39 (Fig 3H–3J). No adverse effects were observed when cells were infected with PLN-A. Overall, these data demonstrate that the PLY-expressing WT *S*. *pneumoniae* (D39) strain can cause severe cardiomyocyte injury and dysfunction *in vitro*, while its PLY- negative isogenic strain cannot. These results strongly suggest that PLY release is responsible for the cardiomyocyte electrical and mechanical dysfunction observed in these experiments.

### Sub-lytic concentrations of PLY induce cardiomyocyte dysfunction *in vitro*

**Fig 4:**
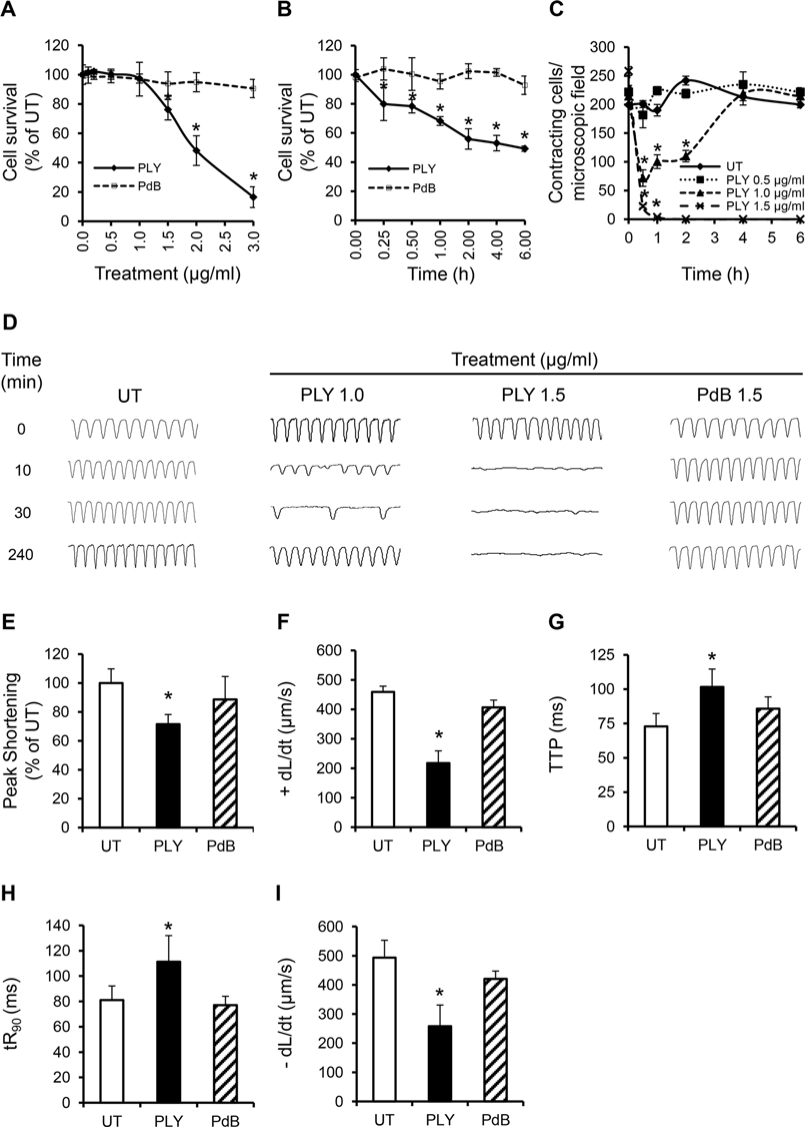
PLY at sub-lytic doses adversely affect cardiomyocyte function *in vitro*. (**A**) Viability of HL-1 cells was assessed 30 min after incubation with increasing concentrations of PLY and PdB using the WST-8 assay. Viability of untreated cells (UT) was set at 100%. Data are presented as Mean±SD. * *p*<0.05 (n = 4). (**B**) Time course of HL-1 cell viability after incubation with 1.5 μg/ml PLY or PdB. *p<0.05 (n = 3). (**C**) Effects of increasing concentrations of PLY on the total number of spontaneously contracting HL-1 cells over time. Data are presented as Mean±SD. *p<0.05 (n = 4). (**D**) Representative traces of cardiomyocyte contraction before and after PLY and PdB treatment (n = 4). (**E-I**) Effects of PLY and PdB (1.0 μg/ml) on Peak Shortening (E), +dL/dt (F), TTP (G), tR_90_ (H) and -dL/dt (I) of HL-1 cells after 30 min treatment are presented as Mean±SD. *p<0.05 (n = 9 from 3 independent experiments).

To investigate whether purified PLY can directly induce cardiomyocyte injury and dysfunction, HL-1 cardiomyocytes were incubated with purified PLY at a range of concentrations. Within 30 min, PLY at 1.5 μg/ml (equal to 150 Haemolytic Units “HU”) significantly reduced cell viability to 76.2±7.0% (Fig 4A, *p* = 0.01). Higher PLY concentrations caused a dose-dependent decline in viability (48.2±10.1% and 16.5±7.1% for PLY at 2 and 3 μg/ml, respectively) (Fig 4A). There was also a time-dependent effect with cell viability continuing to decline to approximately 50% after 2 h incubation with PLY at 1.5 μg/ml (Fig 4B). These results are consistent with those reported by Brown *et al* in which high lytic doses of PLY caused direct cardiomyocyte lysis [16]. However, at lower *sub-lytic* concentrations of PLY, which did not affect cell viability (1 μg/ml PLY at 100 HU), a significant drop in the total number of spontaneously contracting HL-1 cells (214±18/field at 0 min vs 71±14/field at 30 min) (Fig 4C) was observed initially, followed by gradual recovery. At 4 h, recovery in spontaneous contraction was near normal. These data suggest that PLY at sub-lytic concentrations can affect the spontaneous firing of action potentials in cardiomyocyte, but this drop in contractility is *recoverable* over time.

Using Ion-Optix, PLY at 1 μg/ml induced a slow and irregular rhythm in HL-1 cells within a few minutes, with rates of contraction dropping from 4–5 Hz (beat/s) to 2–3 Hz and 1–2 Hz at 10 and 30 min after treatment, respectively (Fig 4D). Rhythm and rate of contraction recovered to near normal levels after around 4 h. In contrast, no recovery was observed with lytic concentrations of PLY at 1.5 μg/ml (Fig 4D). Mechanical indices of contractility at 30 min after exposure to PLY at 1 μg/ml also declined significantly with Peak Shortening reducing to 72±6.6% (Fig 4E, *p* = 0.001) of untreated cells and +dL/dt dropping from 459±19 μm/sto 217±41 μm/s (Fig 4F, *p* = 0.0001). TTP, tR_90_ and -dL/dt were also negatively affected by PLY (Fig 4G–4I), confirming that PLY is responsible for cardiomyocyte contractile electrical and mechanical dysfunction. These data represent novel insights into how pneumococci affect cardiomyocyte function and extend the findings of Fillon *et al* in which only the pneumococcal wall was found to depress cardiomyocyte contractile performance [36].

To investigate if toxicity to cardiomyocytes is due to the lytic activity of PLY alone, a pneumolysin derivative (PdB) [37], with only 0.1% of the haemolytic activity of native wild-type pneumolysin was incubated with HL-1 cardiomyocytes. Fig 4A, 4B and 4D–4I shows that PdB failed to affect the viability, rhythm, rate of contraction or the mechanical parameters of contractility, even at concentrations ≥1.5 μg/ml. These data confirm that membrane pore formation is the mechanism of PLY-induced cardiomyocyte dysfunction.

### Sub-lytic concentrations of PLY induce profound calcium influx leading to cardiomyocyte injury

**Fig 5:**
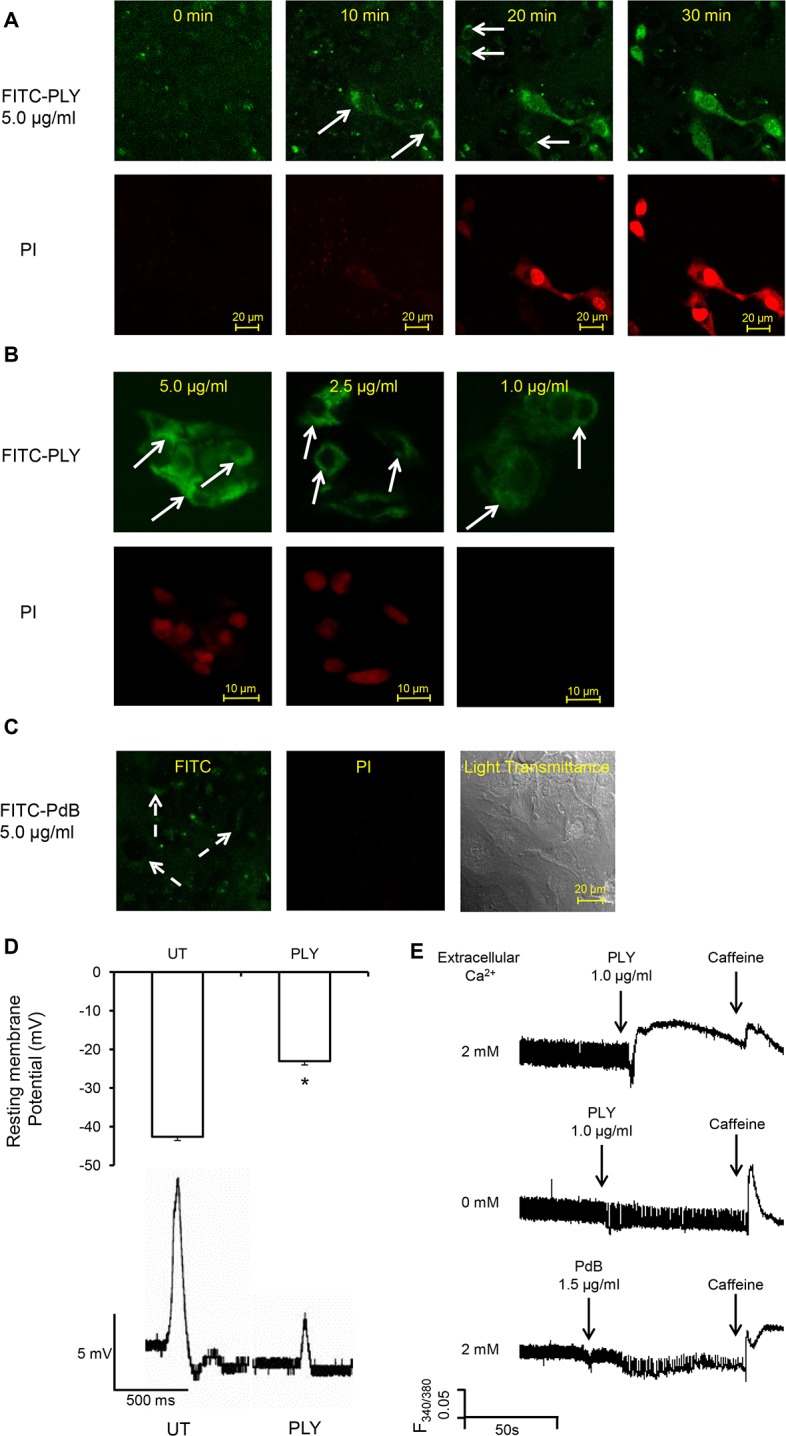
PLY binds to cardiomyocyte membrane and disrupts Ca2+ homeostasis and membrane potential at sub-lytic concentrations. (**A**) HL-1 cells were incubated with 5 μg/ml FITC-PLY and 1.0 μg/ml propidium iodide (PI) to monitor disruption of membrane integrity over time. The localisation of FITC-PLY (green) and PI (red) were recorded using time lapse confocal microscopy (LSM 710, Zeiss) in a maintained environment of 5% CO_2_ at 37°C. Arrows indicate membrane binding of FITC-PLY. Bar = 20 μm (n = 3). (**B**) HL-1 cells were treated with 5, 2.5, or 1 μg/ml FITC-PLY along with 1.0 μg/ml PI for 30 min. Following washing in PBS and fixation in 4% PFA, localisation of FITC-PLY and PI were visualized under the same conditions for comparison. Typical images of each dose are presented (n = 3). Arrows indicate membrane binding of FITC-PLY. Bar = 10 μm. (**C**) HL-1 cells were incubated with 5 μg/ml FITC-PdB and 1.0 μg/ml propidium iodide (PI) to monitor disruption of membrane integrity over time. The localisation of FITC-PdB (green) and PI (red) were recorded as described in (A). Arrows indicate membrane binding of FITC-PdB. Bar = 20 μm (n = 3). (**D**) Membrane potential changes detected in HL-1 cells following exposure to PLY (1 μg/ml). Top panel: histogram showing typical resting membrane potential under untreated (UT) conditions and following exposure to PLY presented as Mean±SD (n = 3).* *p* = 0.01. Bottom panel: Typical action potential of HL-1 cells treated without (UT) or with PLY. (**E**) Changes of [Ca^2+^]_i_ of HL-1 cardiomyocytes with fura-2am as an indicator were recorded using the IonOptix. Typical traces before and after PLY or PdB treatment in presence and absence of extracellular Ca^2+^ are presented. Caffeine (10 mM) was used to induce Ca^2+^ release from sarcoplasmic reticulum stores within cardiomyocytes.

To understand how the pore-forming property of PLY causes cardiomyocyte dysfunction, HL-1 cells were incubated with FITC-labelled PLY. At a high lytic dose (5 μg/ml), propidium iodide (PI) entered cells after PLY binding to the cell membrane (Fig 5A) thereby suggesting PLY cytolytic action and subsequent cell death. However, at sub-lytic concentrations (≤1 μg/ml), no PI was noted inside the cells despite obvious FITC-PLY binding to the cell membrane (Fig 5B). On the other hand, PdB even at a concentration of 5 μg/ml failed to cause any PI influx (Fig 5C) further confirming that the pore-forming property of native PLY is essential for cardiomyocyte injury. We also noticed that PdB had a reduced ability to bind to cardiomyocyte membranes (Fig 5C) as compared to PLY. This may be attributable to the Trp-433 Phe mutation in PdB, which is within the glycan-binding pocket of PLY and can interfere with the ability of the toxin to bind glycans prior to membrane insertion, as recently reported by Shewell *et al* [38]. However, some binding is still detectable with PdB (Fig 5C) suggesting that lack of binding alone does not explain the complete absence of toxicity as compared to PLY. Further investigation demonstrated significant membrane potential changes of HL-1 cardiomyocytes from -42.6±2.0 mV to -23.0±3.4 mV (Fig 5D, upper panel, *p*<0.001) after 30–60 min of exposure to sub-lytic concentration of PLY (1.0 μg/ml). This depolarization of HL-1 cell membrane in response to PLY at sub-lytic concentrations was accompanied by attenuated action potentials (Fig 5D, bottom panel). These data indicate that the plasma membrane permeability changed to a level at which the cell could not maintain its normal membrane potential, despite no reduction in cell viability.

As Ca^2+^ is the key ion regulating cardiomyocyte contractility, changes of intracellular Ca^2+^ to PLY treatment were then monitored to determine these functional effects. Fig 5E (upper panel) and S1-Movie show that sub-lytic concentrations of PLY (1 μg/ml) significantly increased [Ca^2+^]_i_ in cardiomyocytes. This increment was dependent on the concentration of extracellular Ca^2+^ because no significant change in [Ca^2+^]_i_ was observed following PLY treatment when extracellular Ca^2+^ concentration was kept to a minimum (Fig 5E, middle panel). This indicates that PLY-induced changes in [Ca^2+^]_i_ are due to Ca^2+^ influx and not release from intracellular stores. As PdB did not cause significant changes in [Ca^2+^]_i,_ even at 1.5 μg/ml (Fig 5E, lower panel), the membrane-binding and pore-forming properties of PLY appear essential for influx of extracellular Ca^2+^ into cardiomyocytes.

### Reduced Ca^2+^ transient amplitude is a major cause of reduced contractility

**Fig 6:**
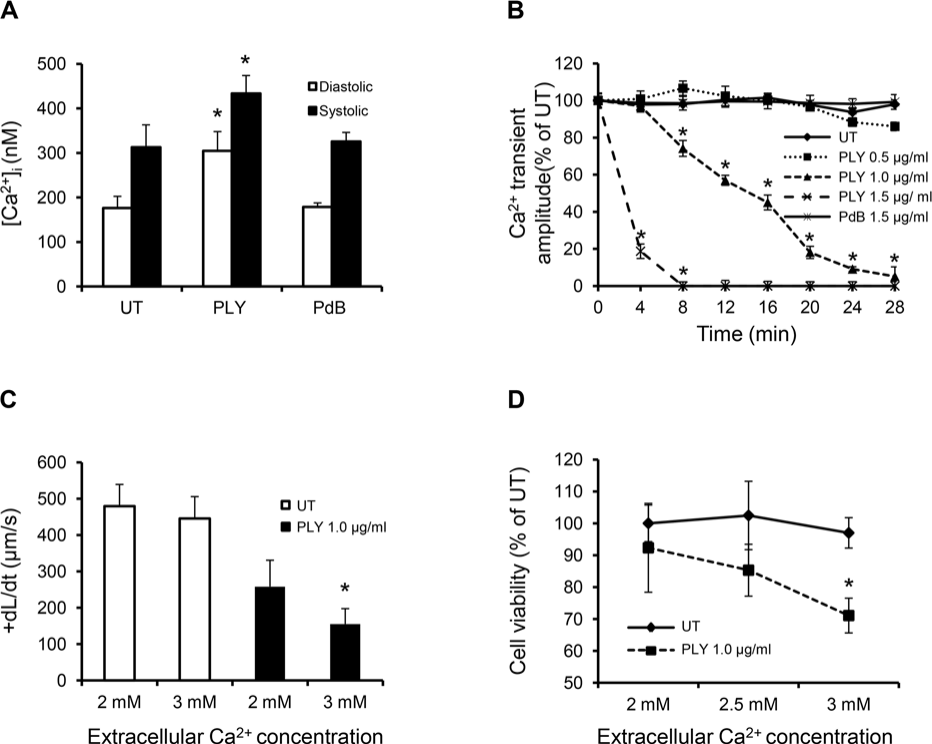
Profound calcium influx induces cardiomyocyte dysfunction and injury in response to sub-lytic PLY. (**A**) Mean±SD of both systolic and diastolic [Ca^2+^]_i_ of HL-cells within 1 min of treatment with PLY/PdB (1.0 μg/ml) from 4 independent experiments are presented. *p<0.05. (**B**) Change in intracellular Ca^2+^ transient amplitude over 30 min in response to different concentrations of PLY. Ca^2+^ transient amplitude at 0 min was set at 100%. Data are presented as Mean±SD (n = 4). (**C**) and (**D**) The effects of changing extracellular Ca^2+^ concentration (in media) of HL-1 cells from 2 to 3 mM on +dL/dt (C) and cell viability (D) in the absence (UT) and presence of 1.0 μg/ml PLY. Mean±SD from 3 independent experiments are presented. * *p*<0.01 when compared to 2 mM extracellular Ca^2+^.

Systolic/diastolic [Ca^2+^]_i_ and the amplitude of intracellular Ca^2+^ transients in cultured HL-1 cardiomyocytes after exposure to PLY were then examined. Systolic and diastolic values of [Ca^2+^]_i_ obtained in untreated cells were consistent with previous reports [39]. Fig 6A shows that diastolic (resting) [Ca^2+^]_i_ increased more dramatically (from 176±26 nM to 304±43 nM, ~42.1% increment) than systolic (peak) [Ca^2+^]_i_ (from 312±52 nM to 433±40 nM ~27.9% increment) following PLY (1.0 μg/ml) treatment to cause a dose and time-dependent reduction in the amplitude of intracellular Ca^2+^ transient (difference between systolic and diastolic [Ca^2+^]_i_) (Fig 6B), which is a key determinant of contractile force. Increasing the concentration of extracellular Ca^2+^ from 2 mM to 3 mM further reduced both contractile performance (Fig 6C) and even converted this sub-lytic dose of PLY(1.0 μg/ml) into a lethal one (Fig 6D), suggesting that enhanced Ca^2+^ influx and subsequent reduction in [Ca^2+^]_i_ transient amplitude is a major cause of reduced contractility and viability. In contrast, PdB did not induce significant changes in [Ca^2+^]_i_ or intracellular Ca^2+^ transient in cardiomyocytes (Fig 6A and 6B). These data strongly support the hypothesis that PLY-induced Ca^2+^ influx (and overload) is the major mechanism of reduced contractility and viability of cardiomyocytes during pneumococcal infection.

### PLY activates pathways associated with sepsis and cardiac dysfunction

Cardiac dysfunction and failure, which is characterized by varying degrees of loss of cardiac function, is frequently associated with activation of signalling events within cardiomyocytes in response to external stimuli [40].

**Fig 7:**
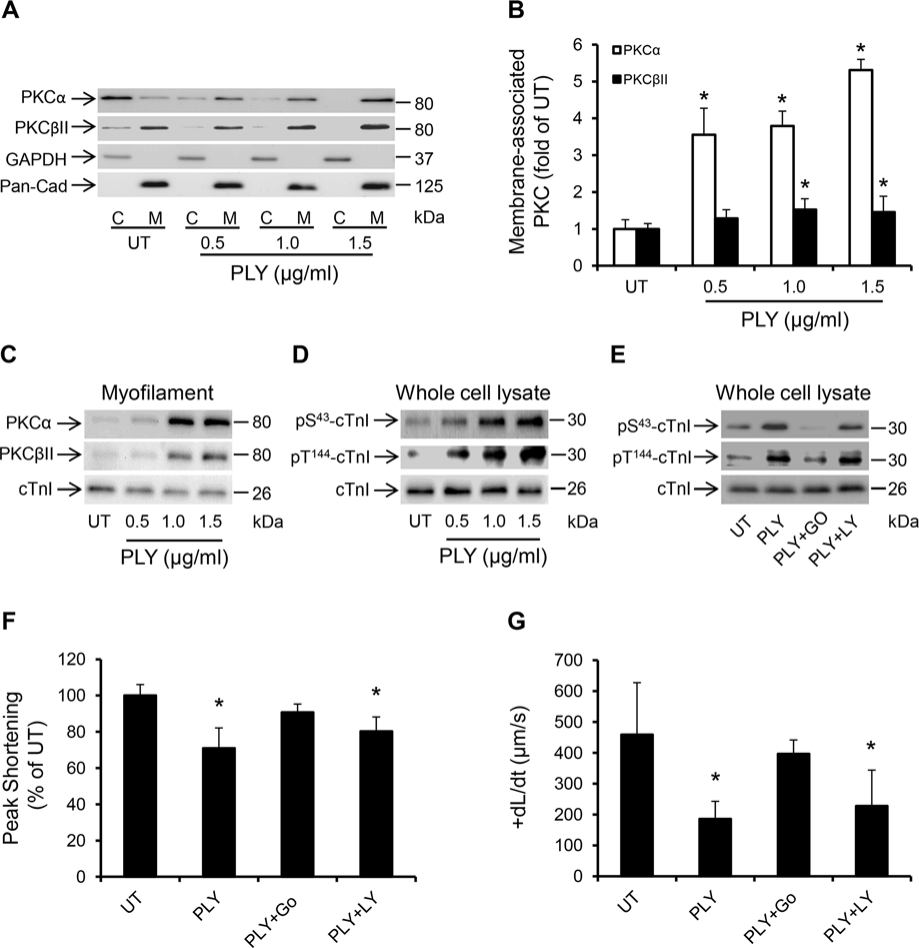
PLY activates the PKCα-cTnI axis in HL-1 cardiomyocytes to depress contractility. (**A**) Representative Western blots and (**B**) a band-quantification histogram showing the distribution of PKCα and PKCβII in the cytosol “C” and membrane “M” fractions of HL-1 cells before (UT) and 30 min after PLY-treatment. GAPDH and Pan-Cadherin were used as markers for “C” and “M” fractions, respectively. Data are presented as Mean±SD (n = 3). * *p*<0.05 compared to UT. (**C-E**) Typical Western blots showing PLY effects on the association of PKCα and PKCβII with the myofilament fraction (C), the phosphorylation of cTnI at the S43 and T144 PKC-dependent phosphorylation sites (D) and the effects of PKCα inhibitor Go6976 (5 nM) and the PKCβII inhibitor LY333531 (10 nM) on PLY (1.0 μg/ml)-induced phosphorylation of cTnI at S43 and T144 phosphorylation sites (E) in HL-1 cells after 30 min of PLY treatment. cTnI was used as endogenous control (n = 3). (**F**) and (**G**) Effects of PLY (1.0 μg/ml) ± Go6976 (5 nM) or LY333531 (10 nM) on Peak Shortening (F) and +dL/dt (G) after 30 min of PLY treatment. Data are presented as Mean±SD (n = 9). *p<0.05 compared to UT.

The PKC-cTnI axis is a well-characterized Ca^2+^-sensitive pathway that modulates the contractile performance of cardiomyocytes by post-translational modifications of myofilament proteins [27]. Activation of this signalling pathway is commonly observed in septic patients with cardiac dysfunction [41,42] but the underlying stimuli remain poorly understood. By measuring the translocation of relevant PKC isoforms from the cytosol to the membrane fraction of HL-1 cardiomyocytes, which is a hallmark sign of their activation [43], we show that PLY caused significant translocation (i.e. activation) of two Ca^2+^-dependent PKC isoforms: PKCα and PKCβII (Fig 7A and 7B). Ca^2+^-sensitive PKCβI was also investigated but found to be expressed at very low levels in cardiomyocyte lysates and not detectable in sub-cellular fractions (S3A Fig). Ca^2+^-insensitive PKC isoforms, such as PKCε, PKCδ (novel PKCs) and PKCζ (atypical PKC) were not affected by PLY (S3A Fig). We further investigated whether PLY enhanced the localization of these two PKC isoforms to cardiac myofilaments, as this would have functional implications [44]. Fig 7C demonstrates that PLY increased the association of PKCα and PKCβII with the Triton X-100-insoluble (myofilament) fraction of HL-1 cells. Successful separation of the myofilament fraction from other cellular compartments using Triton X-100 (1%) is demonstrated in S3C Fig (HL-1 cells) and S3D Fig (murine cardiomyocytes). More importantly, PLY induced dose-dependent phosphorylation of (cTnI) at the PKC-dependent phosphorylation sites (S43 and T144) (Fig 7D), which is known to reduce myofilament responsiveness to Ca^2+^, actin-myosin cross-bridge cycling velocity with eventual effects on contractile force [27,45]. To test the functional consequences of the activation of PKCα and PKCβII on HL-1 cardiomyocytes, these two isoforms were blocked by 1 h incubation with 5 nM PKCα inhibitor Go6976 (reported IC50 for PKCα is 2.3–5 nM) and 10 nM PKCβII inhibitor LY333531 (reported IC50 for PKCβII is 10 nM) [46] prior to PLY (1.0 μg/ml) treatment. Blocking PKCα resulted in significant attenuation of PLY-induced cTnI phosphorylation (Fig 7E) and improvement in Peak Shortening (untreated set as 100%, PLY 70±11%, and PLY+Go6976 90±4%, *p* = 0.012) (Fig 7F) and in +dL/dt (untreated 459±68, PLY 185±45 and PLY+Go6976 397±23 μm/s, *p* = 0.003) (Fig 7G) but PKCβII inhibitor did not show significant effects. Likewise, Go6976 but not LY333531, attenuated PLY effects on TTP, tR_90_ and—dL/dt (S4A–S4C Fig). These data strongly implicate the involvement of the PKCα-cTnI pathway in PLY-mediated cardiomyocyte dysfunction.

**Fig 8:**
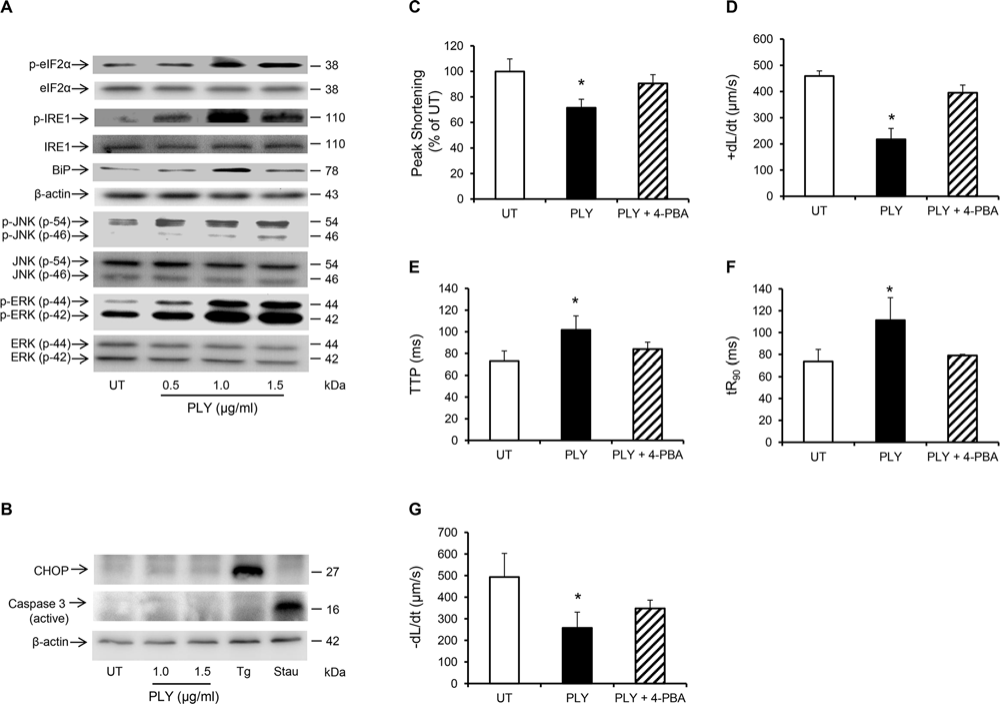
PLY induces endoplasmic reticulum (ER) stress pathway without progressing to apoptosis in HL-1 cardiomyocytes. (**A**) Typical Western blots showing the activation of ER stress markers, (p-elF2α, p-IRE, BiP), JNK and ERK in HL-1 cells 30 min after PLY treatment. (**B**) Representative western blots showing the effect of PLY on the induction of apoptotic markers CHOP and active caspase-3 after 8 hour of treatment. Thapsigargin (Tg 5 μM) and Staurosporin (Stau 10 μM) were used as positive inducers of CHOP and active caspase-3 respectively. (**C-G**) Effects of PLY (1.0 μg/ml) and PLY+ 4-Phenylbutyric acid (4-PBA, an ER stress inhibitor, 10 mM) on Peak Shortening (C), (+dL/dt) (D), TTP (E), tR_90_ (F) and (-dL/dt) (G) of HL-1 cells after 30 min treatment are presented as Mean±SD (n = 9 from 3 independent experiments). * *p*<0.05.

Another signalling pathway that is commonly associated with myocardial dysfunction during invasive infections and sepsis is the Endoplasmic Reticulum (ER) stress pathway [47,48]. It is now understood that disturbance in the excitation-contraction coupling of cardiomyocytes with abnormal Ca^2+^ haemostasis activates ER stress [47], which ultimately precipitates cardiomyocyte dysfunction [49,50]. Furthermore, if the ER stress is not alleviated in a timely manner apoptosis may ensue through the IRE1-JNK-caspase 3 pathway [51] or p-IF2α-CHOP pathway [52]. Treating HL-1 cardiomyocytes with PLY for 30 min activated several ER stress markers including eIF2α, IRE1 and the chaperone protein BiP even at sub-lytic concentrations <1.5 μg/ml (Fig 8A). Furthermore, JNK and ERK were also similarly activated (Fig 8A), however we did not observe activation of the p-38 MAPK. Although it has been previously reported that PLY-induced increment in [Ca^2+^] _i_ can trigger apoptosis in mammalian cells [53] and Brown *et al* have suggested that PLY may be inducing immune-quiescent cardiomyocyte apoptosis [16], PLY failed to induce the ER-stress associated apoptotic marker, CHOP, or the general apoptosis marker, caspase-3 (Fig 8B) (even after 8 hours of treatment with both sub-lytic and lytic doses). These data strongly agree with our *in vivo* findings on the absence of caspase-3 activation in murine hearts exposed to infection with pneumococci (serotype 1, 2 and 6B) or to purified PLY. Nevertheless, alleviating ER stress in HL-1 cardiomyocytes with 10 mM 4-phenyl butyric acid (4-PBA), a chaperon that has previously been shown to efficiently rescue ER stress-induced cardiac dysfunction in various settings [54,55], significantly attenuated the PLY-induced deficit in peak shortening (UT set as 100%, PLY 72±6% vs PLY+4-PBA 91±10%, *p* = 0.041) (Fig 8C) and in +dL/dt (UT 458±43, PLY 217±41 μm/sec vs PLY+4-PBA 395±29 μm/sec, *p* = 0.003) (Fig 8D). Other parameters of contractile function (TTP, tR_90_ and—dL/dt) were also improved by 4-PBA (Fig 8E–8G). These data strongly suggest that ER stress is one of the pathways mediating contractile dysfunction following PLY treatment, although we found no evidence to suggest that ER stress progresses to cardiomyocyte apoptosis in response to PLY.

### PLY activates the same pathways *in vivo*

**Fig 9:**
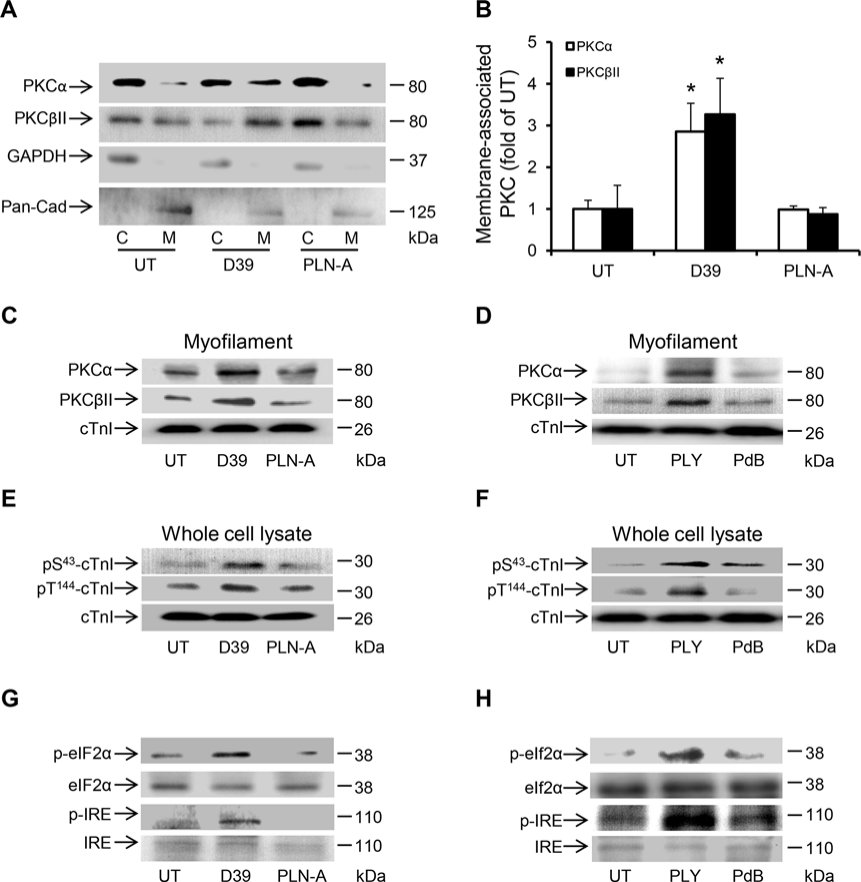
Activation of PKCα-cTnI pathway and ER stress in murine cardiomyocytes exposed to D39 or PLY. (**A**) Representative Western blots and (**B**) a band-quantification histogram showing the distribution of PKCα and PKCβII in the cytosol “C” and membrane “M” fractions of murine cardiomyocytes under untreated (UT), D39- and PLN-A-infection (1x10^6^ CFU) conditions (24 h post-infection). GAPDH and Pan-Cadherin were used as markers for “C” and “M” fractions, respectively. Data are presented as Mean±SD (n = 3). * *p*<0.05. (**C**) and (**D**) Typical Western blots showing the effects of D39/PLN-A (1x10^6^ CFU) infection (C) and PLY/PdB (200 ng/g) i.v. injection (D) on the association of PKCα and PKCβII with the myofilament fraction of murine cardiomyocytes 24 h post injection. cTnI was used as an endogenous control (n = 3). (**E**) and (**F**) Typical Western blots illustrating the phosphorylation of cTnI at the S43 and T144 phosphorylation sites in murine cardiomyocytes following D39/PLN-A infection (E) and PLY/PdB injection (F) (24 h post injection). cTnI was used as an endogenous control (n = 3). (**G**) and (**H**) Typical Western blots showing activation of ER stress markers in murine cardiomyocytes at 24 h post-infection with D39/PLN-A (G) and PLY/PdB (H) (n = 3).

To determine whether *in vivo* cardiac injury was caused by the same mechanisms as demonstrated in cultured cardiomyocytes *in vitro*, the same signalling pathways in murine cardiac muscle cells were examined after exposure to D39 or purified PLY. I.v. injection of D39, but not PLN-A, into mice caused translocation of PKCα and PKCβII to the membrane compartments of murine cardiomyocytes suggesting activation of these Ca^2+^-dependent PKC isoforms (Fig 9A and 9B), whereas Ca^2+^-insensitive PKCs were not affected (S3B Fig), highlighting the pivotal role Ca^2+^ overload in mediating the activation of these detrimental signalling pathways *in vivo* subsequent to pneumococcal infection. Furthermore, i.v. injection of D39 (but not PLN-A) and purified PLY (but not PdB) enhanced the association of PKCα and PKCβII with myofilaments of murine cardiomyocytes (Fig 9C and 9D) and triggered phosphorylation of cTnI at the PKC-dependent phosphorylation residues (S43 and T144) (Fig 9E and 9F). ER stress markers were also activated in murine cardiomyocytes following D39 (but not PLN-A) and PLY (but not PdB) i.v. injection (Fig 9G and 9H). Collectively, these *in vivo* data strongly support our *in vitro* findings on the mechanistic pathways mediating cardiac injury and dysfunction following exposure to pneumococci.

## Discussion

We have shown that *Streptococcus pneumoniae* and its toxin PLY can induce serious cardiac injury and dysfunction. As unexplained acute cardiac events arising during pneumococcal infection are common and associated with an increased risk of fatality, our findings highlight an important potential explanation. We thereby suggest a new mechanism by which cardiac injury occurs and explain how circulating PLY actively drives acute cardiac injuries in the absence of any pneumococcal colonization of the heart. This also explains why effective antibiotic therapy does not immediately reduce cardiac risk as treatment is associated with bacterial lysis and subsequent release of PLY, which would in turn disrupt normal cardiac function. In our model of IPD, the consistency of our findings using three different pneumococcal serotypes (serotype 1, serotype 2 and 6B) strongly suggest that blood circulating PLY is the major mediator of cardiomyocyte injury, dysfunction and death. We show that intravenous injection of PLY into mice caused substantial release of cardiac troponins; biomarkers of cardiomyocyte injury, into the circulation as quickly as 12 hours post infection, with peak levels at 24 hours post injection, suggesting early and significant cardiomyocyte injury.

Our findings complement and further extend on a recent study by Brown *et al* which provided evidence for myocardial microlesion formation subsequent to pneumococcal translocation across the myocardial vasculature by pneumococcal-endothelial cell interactions mediated by the CbpA-Laminine receptor (LR) and ChoP-Platelet activating factor receptor (PAFR) [16]. Brown *et al* showed that by use of anti-PLY antibodies, myocardial microlesion formation could be prevented. However, when purified PLY was directly administered by retro-orbital injection into mice, this failed to induce myocardial microlesions suggesting that at least in their model of infection, cardiomyocyte damage occurred remotely possibly via release of pneumococcal toxic mediators such as PLY or other pneumococcal factors such as capsular polysaccharide subsequent to pneumococcal translocation to the myocardium [16]. Hence, a direct role for PLY was not identified as purified PLY failed to induce microlesions in the myocardium despite causing major inflammation and sloughing of the endothelial cells in the myocardial vasculature [16]. In contrast, our findings indicate that blood circulating PLY is *key* to cardiomyocyte damage *in vivo*, regardless of pneumococcal translocation or colonization of the myocardium. This is supported by our demonstration that i.v. injection of wild type whole (PLY-expressing) pneumococci, but not PLY-deficient pneumococci, trigger substantial release of cardiac troponins into the circulation. Furthermore, we demonstrate a similar elevation of circulating cardiac injury biomarkers in experiments performed with a PLY-expressing but CbpA-deficient strain, which was unable to adhere to the vascular endothelium or translocate to the myocardium. This finding further indicates that myocardial injury can happen in the absence of direct pneumococcal translocation or infection of the myocardium. It must be highlighted of course that our model of IPD is distinct from that used by Brown *et al* [16]. The bolus injection of pneumococci into the blood stream used in our model could have resulted in rapid disease progression and different host immune responses that may have prevented the development of myocardial infective foci. However, in terms of clinical relevance, our findings are in line with several post-mortem studies performed on patients dying of fulminant pneumococcal sepsis, which showed pneumococci in the blood stream and lungs but documented no myocardial pneumococcal colonization or gross myocardial abnormalities [56–58]. This is in contrast to the recent finding by Brown *et al* documenting myocardial vacuolar lesions in 2 out of 9 (22%) adult human autopsies succumbing to IPD [16]. Finally, a potential further difference, is that experiments with purified PLY were performed at different concentrations, via different routes of administration (intravenous vs. retro-orbital) with different outcomes (cardiac troponin release vs myocardial microlesion formation) examined in our study compared to Brown *et al* [16].

A key finding of our study was the potential correlation between elevated cardiac troponins in mice and their increased mortality. Mice with high levels of circulating cardiac troponins at 24 hours post exposure to PLY-expressing pneumococci were significantly less likely to survive. This is a significant finding as it is well established that elevated cardiac troponins strongly predict and correlate with cardiac muscle dysfunction as well as prognosis of patients [12,59,60]. This confirms that PLY plays a pivotal role in cardiomyocyte damage during pneumococcal infections *in vivo* and also suggests that circulating PLY may contribute to the elevated troponin levels frequently detected in patients with CAP [7]. The clinical implications of our findings are supported by a previous study documenting the high prevalence (41%) of pneumococcal infections in septic patients admitted to the intensive care unit with elevated cardiac troponin levels [61]. Importantly, we provide a novel therapeutic application for targeting circulating PLY using engineered liposomes that sequester circulating PLY [28] to attenuate cardiac injury and the elevation of cardiac troponins during IPD. This has not been shown before and clearly demonstrates that therapeutic interventions which neutralise circulating PLY and its action, may have significant clinical benefit to patients with pneumococcal disease.

We also demonstrate the ability of pneumococci to directly damage and kill cardiac muscle cells, via PLY release, irrespective of pneumococcal colonization of the myocardium. This is supported by the observations that (1) PLY-deficient pneumococci were completely attenuated in their ability to induce cardiomyocyte dysfunction, (2) using antibiotics in culture to lyse PLY-expressing pneumococci (and therefore release PLY [62,63]), further exacerbated the toxic effects on cardiomyocytes, an outcome which was absent when antibiotics were used with PLY-deficient pneumococci, (3) the use of PLY-sequestering liposomes in the circulation of mice significantly attenuated cardiac injury subsequent to infection with WT PLY-expressing pneumococci and (4) i.v. injection of purified PLY caused substantial increases in circulating cardiac troponins and myocardial inflammation. We also show strong inflammatory cell infiltration into the myocardial tissue using three different WT pneumococcal serotypes (1, 2 and 6B). This observation remained consistent when we used PLY-expressing but CbpA-deficient pneumococci or purified PLY injections, suggesting that such changes are independent of CbpA-mediated pneumococcal translocation to the myocardium. Such strong inflammatory changes are typically associated with necrotic cell death and this is further supported by the absence of cardiomyocyte apoptosis in our IPD model. Indeed, PLY failed to induce apoptotic markers, such as CHOP or caspase-3, even after prolonged treatment for up to 8 hours, although it did induce ER stress and MAPK pathways (IRE1, p-eIF2α, JNK) that can turn pro-apoptotic in certain cases. Importantly though, we were unable to identify activation of caspase-3 in heart sections from mice dying of IPD between 24 and 96h post infection with pneumococci (serotype-2, serotype-1 6B and CbpA-deficient strains) or post PLY i.v. injection. These findings suggest that apoptosis is unlikely to be the mode of death of cardiomyocytes in these circumstances and that a necrotic cellular death is more plausible, especially given the intense inflammatory cell infiltration into the myocardium and cardiac troponin release without gross myocardial abnormality in our model of IPD.

The major molecular mechanism of PLY cytotoxicity is in its ability to disrupt the cell membrane by binding to cholesterol and causing pore formation. We established the importance of the membrane-binding and pore-forming abilities of PLY to pneumococcal-induced cardiomyocyte dysfunction by demonstrating that a PLY-mutant (pneumolysin toxoid B “PdB”), with significantly reduced membrane-binding and pore forming abilities [37], can still bind to cardiomyocyte membranes but without causing functional consequences. Wild type PLY binds to cardiomyocyte membrane to induce cell death at high concentrations (≥1.5 μg/ml in our experiments). More interestingly, however, we report here that at *sub-lytic* doses, PLY induces a range of cellular events with adverse functional implications including depolarization of the cellular membrane, calcium overload and activation of signalling pathways with known detrimental effects to cardiomyocyte function and wellbeing. These data are consistent with a mechanism whereby sub-lytic concentrations of PLY can disrupt plasma membrane integrity and increase its permeability to ions, whilst high concentrations of PLY can cause large pore formation, cellular lysis and cell death. Indeed, this finding is in agreement with recent reports that PLY can generate multiple conductance (different size) pores in membranes depending on its concentration [64,65]. Significant cardiomyocyte depolarization in various settings is known to be associated with reduced excitation and conduction velocity and may contribute to arrhythmogensis [66]. In our experiments, cardiomyocyte membrane potentials were depolarized by ~50% in response to sub-lytic PLY concentrations. We also observed disturbance of spontaneous rhythm in cultured cardiomyocytes after exposure to PLY, but interestingly this effect may be spontaneously recoverable. The abnormal permeability to ions and resultant membrane depolarization induced by PLY may be a major mechanism of arrhythmia, which frequently occurs in patients with pneumococcal infection. Our observation of significant membrane depolarization and extracellular Ca^2+^-dependent increases in [Ca^2+^]_i_ strongly indicate PLY-induced permeability changes as exemplified by a strong Ca^2+^ influx, an effect well-known to mediate PLY toxicity in various mammalian cells [19,20,67]. These PLY induced effects have not been reported before for cardiomyocytes or associated with cardiac injury and dysfunction.

Ca^2+^ overload is a major mediator of cardiomyocyte contractile dysfunction, injury/death, as well as arrhythmias [25,68–70]. Reduction of Ca^2+^ transient amplitude and activation of Ca^2+^ sensitive signalling pathways, such as PKCα-cTnI, and induction of ER stress both *in vitro* and *in vivo* may play an important role in reducing contractile performance. Previous studies have documented that PKCα activation and enhanced association with cardiac myofilaments convey negative effects on contractility [44,45,71]. Our finding that PLY-induced cTnI phosphorylation is mainly PKCα-dependent is therefore of significant relevance and in line with previous clinical studies [45,71,72] highlighting the potential of PKCα and ER stress inhibition as therapeutic options for cardiac dysfunction. Future studies may be tailored to investigate the potential development of arrhythmias and myocardial contractile dysfunction *in vivo* during IPD and the impact of such complications on survival using electrocardiogram (ECG), echo and intra-ventricular catheters. Such studies will be crucial to exploring the efficacy of liposome-based toxin sequestration therapy [28] as well as PKCα [73,74] and ER stress [72] inhibition to alleviating cardiac injury during IPD.

**Fig 10:**
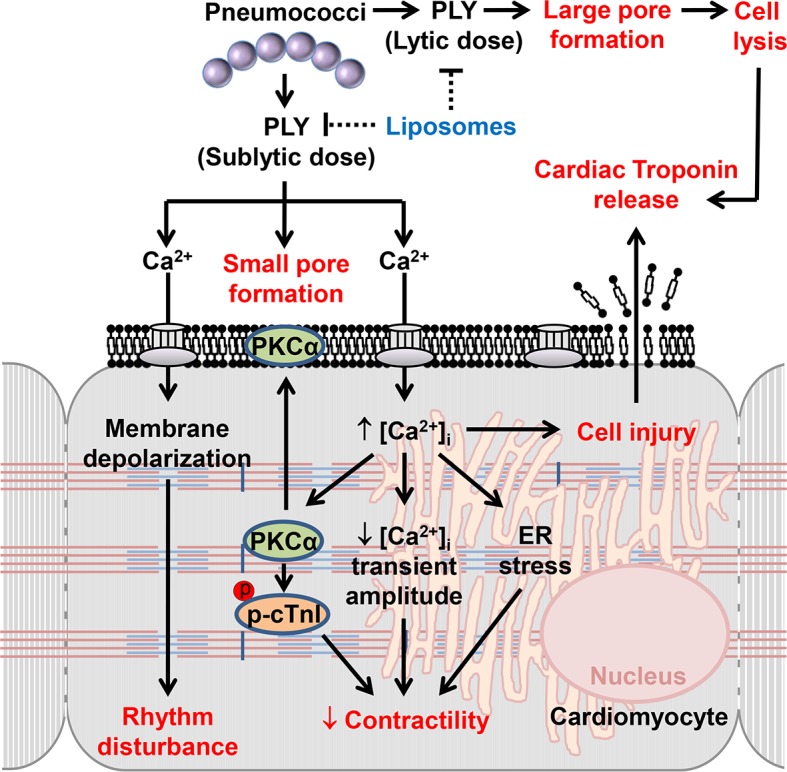
The mechanisms and effects of PLY on cardiomyocytes. High lytic concentrations of PLY induce large pore formation to lyse cells. However, *sub-lytic* concentrations of PLY bind cellular membrane to induce smaller pores thereby triggering profound Ca^2+^ influx into cardiomyocytes. The resulting abnormal increment in intracellular Ca^2+^ concentration [Ca^2+^]_i_ causes significant membrane depolarization, activation of detrimental signalling pathways (e.g. PKCα-cTnI axis, ER stress) and reductions in the Ca^2+^ transient amplitude to cause rhythm disturbance and depression in contractile force. Ultimately, Ca^2+^ overload causes cellular injury which may account for cardiac troponin leakage from cardiomyocytes into the circulation. Toxin-sequestering liposomes offer a potential novel therapeutic intervention against the toxic effects of circulating PLY.

Collectively, our *in vitro* studies of cardiomyocytes and *in vivo* studies of invasive pneumococcal infection demonstrate that pneumococci are toxic to cardiomyocytes and that the pneumococcal toxin (PLY), is the major mediator of cardiomyocyte damage, dysfunction and death. The underlying molecular mechanisms, as summarized in Fig 10, provide a novel explanation for cardiomyocyte injury during invasive pneumococcal infection, which often precipitates life-threatening cardiac complications. Depending on its concentration, PLY can mediate extensive cardiomyocyte damage, lysis and death or a wide range of deleterious signalling events driven by pore-formation and calcium overload. Furthermore, our findings open a novel translational pathway of utilizing circulating PLY as a biomarker to predict acute cardiac injury development in patients and for targeting PLY specifically during invasive pneumococcal disease to reduce the risk of development of cardiac complications. Novel translational strategies and techniques to accurately detect and quantify circulating levels of PLY in patients with IPD are therefore exigent. Indeed, we demonstrate here the therapeutic potential of using specifically targeted toxin sequestration therapy against PLY *in vivo* [28], to protect against cardiac injury during IPD.

## Materials and Methods

### Preparation of bacteria and pneumolysin

*S*. *pneumoniae* serotype 2, strain D39 (NCTC 7466), was obtained from the National Collection of Type Culture, London, UK. Its isogenic pneumolysin-negative mutant, PLN-A, was generated by insertion duplication mutagenesis, as previously described [29]. The ΔPLY strain was generated by *ply*-deletion in D39, as described previously[30]. The CbpA deletion generated on the serotype 2 (D39) background was a kind gift from Prof. Jeremy Brown (UCL, UK). Serotype 6B (sequence type 138) and serotype-1 (sequence type 300) were both clinical isolates taken from patients with sepsis. Serotype-1 (sequence type 306) was a clinical isolate from a patient with emphysema. Briefly, bacteria from bead stocks were streaked onto blood agar and grown overnight at 37°C. Pneumococci were identified by presence of a zone of alpha haemolysis around each colony and a zone of inhibition around an optochin disc. A sweep of colonies was inoculated into brain heart infusion (BHI) broth and grown statically overnight at 37°C. The next day a small volume of overnight growth was sub-cultured into BHI 20% (v/v) fetal calf serum and grown statically for 4–6 hours until mid-log phase growth (OD500 1.4), at which point broth was divided into 500μl aliquots and stored at -80°C in BHI broth containing 20% (v/v) fetal bovine serum for no more than 6 months until use [75]. Prior to use in experiments, two stock aliquots were thawed at room temperature and plated on blood agar by Miles and Misra dilution to quantify stock concentrations (CFUs). Recombinant wildtype PLY and its derivative PdB (0.1% haemolytic activity of wild type PLY) were expressed in *E*. *coli* and purified, as previously described [22]. After purification, the toxin was passed 3 times through an EndoTrap endotoxin removal column (Profos AG, Germany) after which LPS was undetectable using the PyroGene Recombinant Factor C assay (Lonza; detection limit 0.01 EU/ml). PLY and PdB purities were >97% as determined by SDS-PAGE electrophoresis. Haemolytic activity of PLY (and PdB) was determined as previously described [37] by serial dilution of the toxin into sheep erythrocytes. Unless otherwise stated, the specific haemolytic activity of PLY was 100,000 HU/mg.

### Murine *in vivo* experiments

7–12 week old, age-matched, female MF1 mice (Charles River, UK) were used. Bacteria (1x10^6^ CFU D39, PLN-A, ΔPLY, serotype-1, 6B or CbpA knockouts in 50 μl saline) or protein (200–400 ng/g PLY or PdB 400 ng/g in 50 μl saline) were administered through tail vein. Mice were monitored for signs of disease, pain score was determined using the scheme of Morton [76] and animals were euthanized if they reached the experimental endpoint. Liposomes 100 mg/kg of a 1:1 mixture of Cholesterol:Sphingomyelin (66 mol/% cholesterol) and Sphingomyelin only liposomes were injected i.v. 30 min following infection with D39. Blood samples were withdrawn from the tail vein at periodic intervals post-injection for assessment of plasma cardiac troponin levels. Briefly, blood samples were withdrawn from the tail vein before and 12 and 24 h after i.v. injection of D39, PLN-A, serotype-1 (ST300 and ST306), serotype 6B, ΔPLY pneumococci and CbpA-knockout pneumococci (1x10^6^ CFU in 50 μl PBS) or PLY (200 ng/g)/PdB (400 ng/g). 3 μl of each plasma sample was mixed with SDS lysis buffer, boiled at 100°C for 10 min and cardiac troponins were measured by Western blotting using specific antibodies against cTnI (Abcam) and cTnT (Cell signalling). cTnI was quantified by running known concentrations of recombinant cTnI together with the samples.

Hearts were removed at the end of the experiments and LV tissues were homogenized and lysed for signalling pathway analysis by Western blotting. All animal experiments were performed at the University of Liverpool with the approval of the Local Animal Welfare and Ethics Committee and under UK Home Office Project Licence.

### Pathological examination of murine heart sections

Hematoxylin and eosin (H&E) and immunohistochemical staining with anti-active caspase-3 (abcam) (1:10) or anti-serotype 1, 2, 4, 5 and 18 pneumococcus antiserum (SSI Diagnostica) (1:500) were carried out as described previously [77].

### HL-1 culture and viability assessment

Murine HL-1 cardiomyocytes were a kind gift from Prof WC Claycomb (Louisiana State University Medical Centre, New Orleans, LA). Cells were cultured in Claycomb medium, as previously described [34]. When fully confluent, cells showed spontaneous contraction at a rate of 5–6 Hz at 37°C. Viability of cardiomyocytes was assessed using the WST-8 assay (Enzo Life Sciences). Briefly, 5x10^4^ cells were seeded into each well of a 96-well plate and grown until fully confluent. After treatment, the medium was changed and 10 μl of WST-8 dye was added to 100 μl of medium/well, followed by further incubation for 2 h. OD450 nm against reference OD650 nm was measured to represent viable cells.

### Counting number of spontaneously contracting cardiomyocytes

A 35 mm dish containing spontaneously contracting cells was mounted into the stage of an inverted microscope and temperature was maintained at 37°C. The total number of contracting cells was counted and recorded before treatment (0 h). 4 random microscopic fields were selected and mean±SD were calculated. Cells were then treated with either medium only, different concentrations of PLY or pneumococci (D39/PLN-A) and the counting process was repeated at specified time points.

### Confocal microscopy

PLY was labelled with FITC using FluoroTag FITC Conjugation Kit (Sigma). HL-1 cells cultured in a 35 mm glass bottom dish were incubated with 5 μg/ml FITC-PLY and 1 μg/ml PI for monitoring cell damage over time. Localisation of FITC-PLY and PI were recorded using time lapse confocal microscopy (LSM 710, Zeiss) in a maintained environment of 5% CO_2_ at 37°C. To investigate the effect of different concentrations of FITC-PLY on cardiomyocyte damage, HL-1 cells were seeded on 8 chamber culture slides (BD Falcon) in medium containing 10% FBS until confluent. Cells were then treated with 5, 2.5, 1, or 0.5 μg/ml FITC-PLY along with 1 μg/ml PI for 30 min. Following washing in PBS and fixation in 4% PFA, FITC-PLY and PI distribution were visualized using a Zeiss LSM 510 confocal microscope.

### Measurement of membrane potential

HL-cells were plated into the tissue chamber of a Nikon E600FN upright microscope and superfused with medium at 37°C. For PLY exposure, the peptide was added to the superfusing medium for up to an hour. Membrane potentials were recorded with sharp electrodes (>50MΩ filled with 1M KCl) using an Npi SEC 05LX amplifier as described previously [78]. Data was filtered at 1 kHz and acquired to PC computer using an Axon Digidata 1200 interface at 5 kHz. Data analysis was with Dempster’s WinEDR software (University of Strathclyde, UK).

### HL-1 cardiomyocyte contractility and Ca^2+^ flux

HL-1 cardiomyocyte contractility and Ca^2+^ flux were recorded using a video edge-recognition system (IonOptix, MyoCam-S, Dublin, Ireland), as previously described [39,79]. Briefly, 10^6^ cells were seeded into 35 mm glass-bottom tissue culture dishes (Corning) and cultured at 37°C and 5% CO_2_ until approximately >75% of the cells were spontaneously contracting (2–3 days). Experiments were performed on spontaneously contracting as well as on paced cardiomyocytes (at 5 Hz) and comparable results were obtained. Cells that showed irregular contractions or no response to electrical stimulation were excluded. After one minute of recording, treatment was gently perfused across the cells and contractility was recorded for a period of 30 min. In experiments with chemical inhibitors (Go6976 or LY333531 (Sigma) or 4-Phenylbutyric acid (4-PBA, Calbiochem), these were pre-incubated with the cells for 1 h prior to PLY treatment. All recordings were performed at 37°C. Mechanical parameters of contractility (Peak Shortening, TTP, tR_90_, +dL/dt and -dL/dt) were analyzed using the Ion-Wizard 6.0 software.

For Ca^2+^ flux recording, Fura-2AM (Invitrogen) (10 μM) was incubated with cells for 45 min, washed and incubated at 37°C for a further 10 min in fully supplemented Claycomb medium to allow the dye to de-esterify. Cells were then mounted into an inverted microscope and intracellular calcium flux was recorded continuously using an Optoscan monochromator fluorescence photometry (Cairn Research, Faversham, Kent, UK) for 30 min after treatment. Fluorescence signals were elicited by alternate excitations with respective wavelengths of 340 and 380 nm at 250 Hz and recorded at 510 nm through a photomultiplier tube. In order to quantify [Ca^2+^]_i_, cells were treated with (10 mM) Caffeine (Sigma,UK) to get the maximum (R_max_) and (25 mM) EDTA to get the minimum (R_min_) intensity. [Ca^2+^]_i_ = Kd*(R-R_min_)/(R_max_-R)*Sf2/Sb2, where Kd is the Fura-2AM dissociation constant (set at 225 nM). The values of Sb2 and Sf2 correspond to fluorescence excited by the denominator wavelength under conditions of saturating calcium levels (bound state) and in the absence of calcium (calcium-free state) respectively.

### Preparation of cell lysates and subcellular fractionations

For cell lysates, HL-1 cells were directly lysed using SDS lysis buffer, whilst mouse left ventricular (LV) tissues were homogenised in the lysis buffer. Subcellular fractionation was performed as previously described with slight modifications. In brief, HL-1 cells or homogenized murine LV tissues in homogenization buffer (25 mM Tris, 2 mM EDTA, 10% Glycerol) were shredded by passing through 31 gauge needles. The nuclear fraction was removed by centrifugation at 1,000 g for 5 min and resultant supernatant was centrifuged at 100,000 g for 45 min to separate cytosolic (supernatant) and membrane (pellet) fractions. For myofilament fraction purification, HL-1 and murine cardiomyocytes were incubated with 1% Triton X-100 in ice-cold PBS with shaking for 10 min. The resultant supernatant, mainly containing cytosolic and membrane fractions, was transferred to a separate tube. This process was repeated 3 times and the remaining Triton X-100-insoluble (myofilament) fraction was lysed with SDS lysis buffer and subjected to Western blotting.

### Statistical analysis

Statistical significance between multiple groups was determined by one-way ANOVA test (LSD post-hoc). Student’s t-test was used if the comparison involves two groups only. Differences were considered significant when *p* values were <0.05. Linear correlation analysis between circulating cTnI levels and blood pneumococcal CFUs was performed using Spearman correlation test.

## Supporting Information

S1 Fig PLY-sequestering liposomes attenuate cardiac injury during IPD. (**A**) Blood CFU counts of D39 and PLN-A after 12 and 24 post infection with 10^6^ CFU intravenously. (**B**) A representative Western blotting showing profound reduction in circulating cardiac injury biomarkers, cardiac troponin I and T (cTnI and cTnT) in the circulation of mice infected with WT D39 pneumococci by the i.v. administration of engineered liposomes (lipo) 30 min after the D39 (1x10^6^ CFU) injection. (n = 3).
(TIF)

S2 Fig PLY-expressing CbpA-deficient strains, induce cardiac injury and inflammatory cell infiltration into the myocardium. (**A**) Representative Western blots showing circulating cTnI and cTnT in murine plasma following i.v. injection of ΔCbpA (n = 4) (1x10^6^ CFU). (**B**) Histo-pathological examination of murine hearts after infection with ΔCbpA. (*a*) H&E representative images of murine heart sections under x4 magnification showing absence of gross myocardial pathology. (*b*,*c*) Immunohistochemistry images showing absence of pneumococcal capsule staining (*b*) and absence of active caspase-3 staining (*c*) in hearts from mice infected with ΔCbpA.
(TIF)

S3 Fig PLY effects on Ca^2+^-insensitive PKC isoforms. (**A**) and (**B**) Representative Western blots illustrating the cytosol “C” to membrane “M” distributions of PKCε, PKCδ (novel PKCs) and PKCζ (atypical PKC) following PLY treatment of HL-1 cardiomyocytes (A) and in murine cardiomyocytes intravenously injected with D39/PLN-A (1x10^6^ CFU) (24 h post-infection) (B). (n = 4). (**C**) and (**D**) Representative Western blots illustrating successful separation of the myofilament “Myo” (Triton-insoluble) fraction from the cytosol-membrane “CM” (Triton-soluble) fraction of HL-1 cardiomyocytes (C) and murine cardiomyocytes (D). GAPDH, Pan-Cad and cTnI were used as markers for “C”, “M” and “Myo” fractions, respectively (n = 4).
(TIF)

S4 Fig PLY activates the PKCα-cTnI axis in cardiomyocytes to depress contractility. Effects of PLY (1 μg/ml) ± Go6976 (5 nM) or LY333531 (10 nM) on time to peak (TTP) (**A**), time to 90% re-lengthening (tR_90_) (**B**) and the maximum velocity of re-lengthening (-dL/dt) (**C**) of HL-1 cells after 30 min treatment. Data are presented as Mean±SD. *p<0.05 ANOVA test. (n = 9).
(TIF)

S1 Movie PLY induces increased [Ca^2+^]_i_ and disturbed Ca^2+^ dynamics in HL-1 cardiomyocytes. Time-lapse confocal microscopy was performed on HL-1 cardiomyocytes loaded with Ca^2+^ probe Fluor-4AM (green fluorescence). PLY 1 μg/ml was added after basal Ca^2+^ waves have been recorded. In this movie, the first two Ca^2+^ waves serve as an untreated control, whereas the following waves are affected by PLY treatment. PLY clearly increased the intensity and frequency of Ca^2+^ waves (green fluorescence). In addition, more irregularity (loss of synchronization) among cells can be seen following PLY treatment.
(AVI)
