# Supplementary figures and images for: Circulating Pneumolysin Is a Potent Inducer of Cardiac Injury during Pneumococcal Infection
Source: PLoS Pathog. 2015 May 14;11(5):e1004836. doi: 10.1371/journal.ppat.1004836 (PMC4431880; doi:10.1371/journal.ppat.1004836)

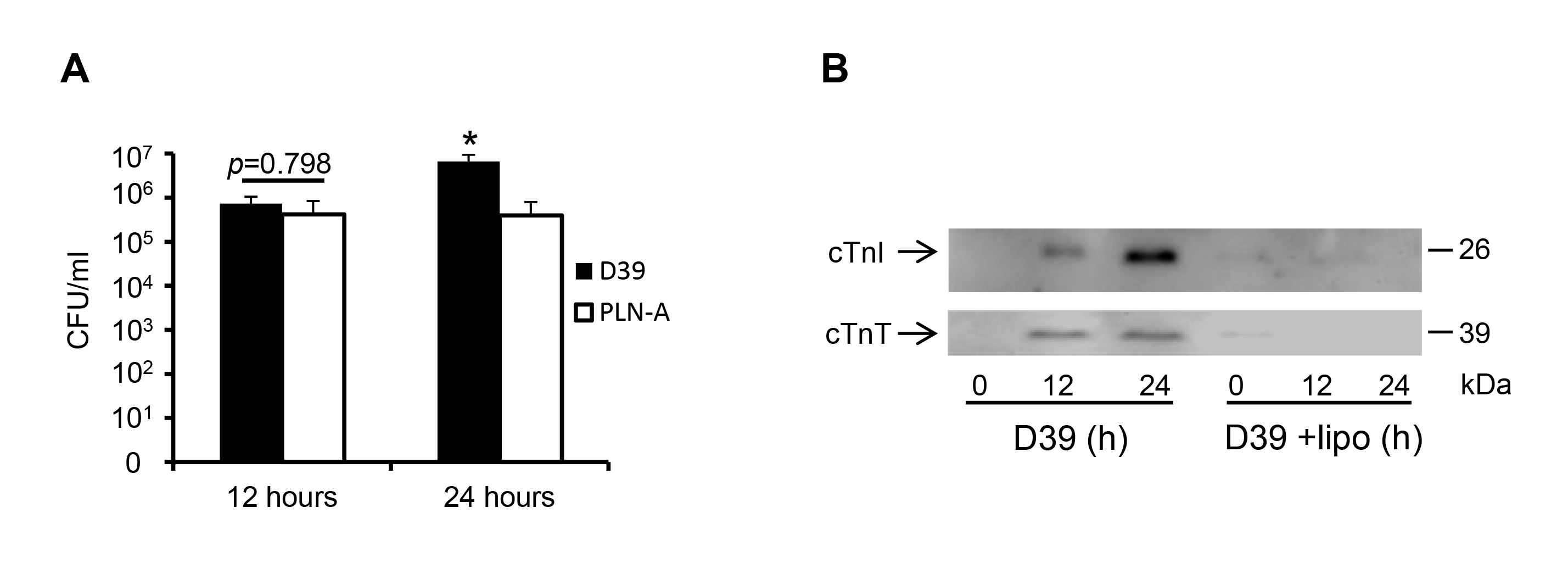

Supplement: S1 Fig — (A) Blood CFU counts of D39 and PLN-A after 12 and 24 post infection with 106 CFU intravenously. (B) A representative Western blotting showing profound reduction in circulating cardiac injury biomarkers, cardiac troponin I and T (cTnI and cTnT) in the circulation of mice infected with WT D39 pneumococci by the i.v. administration of engineered liposomes (lipo) 30 min after the D39 (1x10^6^ CFU) injection. (n = 3). (TIF) [file ppat.1004836.s001.tif]

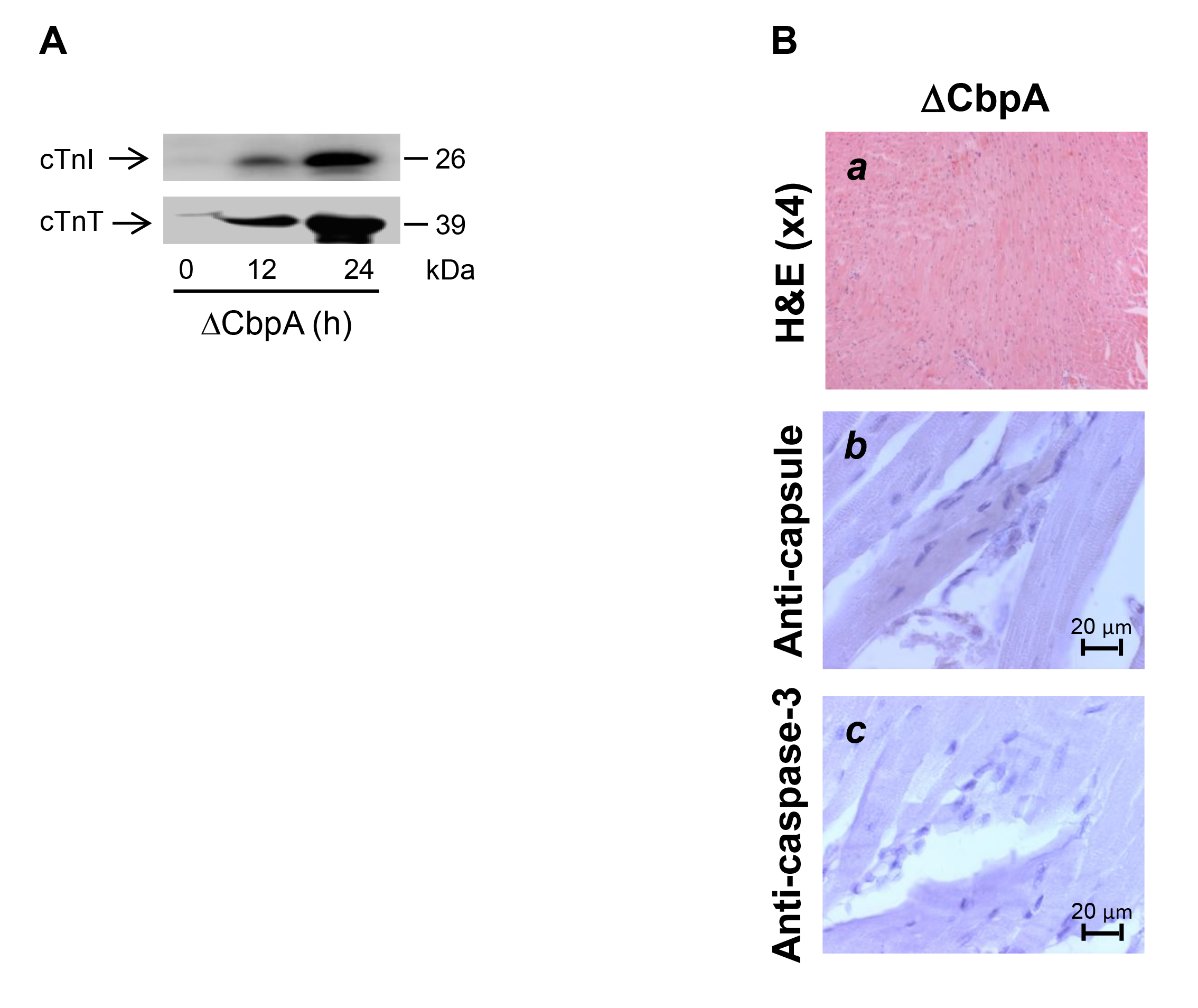

Supplement: S2 Fig — (A) Representative Western blots showing circulating cTnI and cTnT in murine plasma following i.v. injection of ΔCbpA (n = 4) (1x10^6^ CFU). (B) Histo-pathological examination of murine hearts after infection with ΔCbpA. (a) H&E representative images of murine heart sections under x4 magnification showing absence of gross myocardial pathology. (b,c) Immunohistochemistry images showing absence of pneumococcal capsule staining (b) and absence of active caspase-3 staining (c) in hearts from mice infected with ΔCbpA. (TIF) [file ppat.1004836.s002.tif]

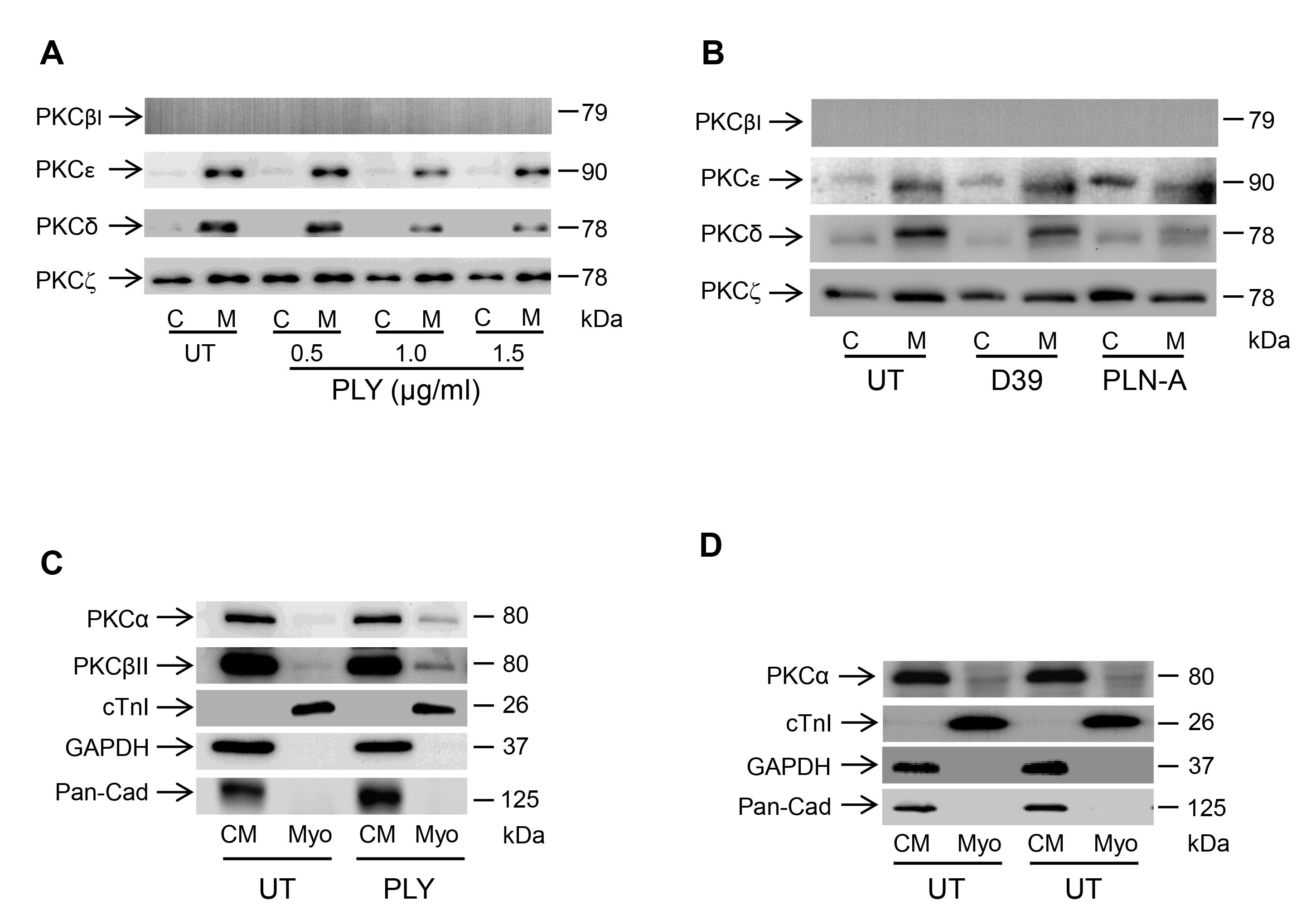

Supplement: S3 Fig — (A) and (B) Representative Western blots illustrating the cytosol “C” to membrane “M” distributions of PKCε, PKCδ (novel PKCs) and PKCζ (atypical PKC) following PLY treatment of HL-1 cardiomyocytes (A) and in murine cardiomyocytes intravenously injected with D39/PLN-A (1x10^6^ CFU) (24 h post-infection) (B). (n = 4). (C) and (D) Representative Western blots illustrating successful separation of the myofilament “Myo” (Triton-insoluble) fraction from the cytosol-membrane “CM” (Triton-soluble) fraction of HL-1 cardiomyocytes (C) and murine cardiomyocytes (D). GAPDH, Pan-Cad and cTnI were used as markers for “C”, “M” and “Myo” fractions, respectively (n = 4). (TIF) [file ppat.1004836.s003.tif]

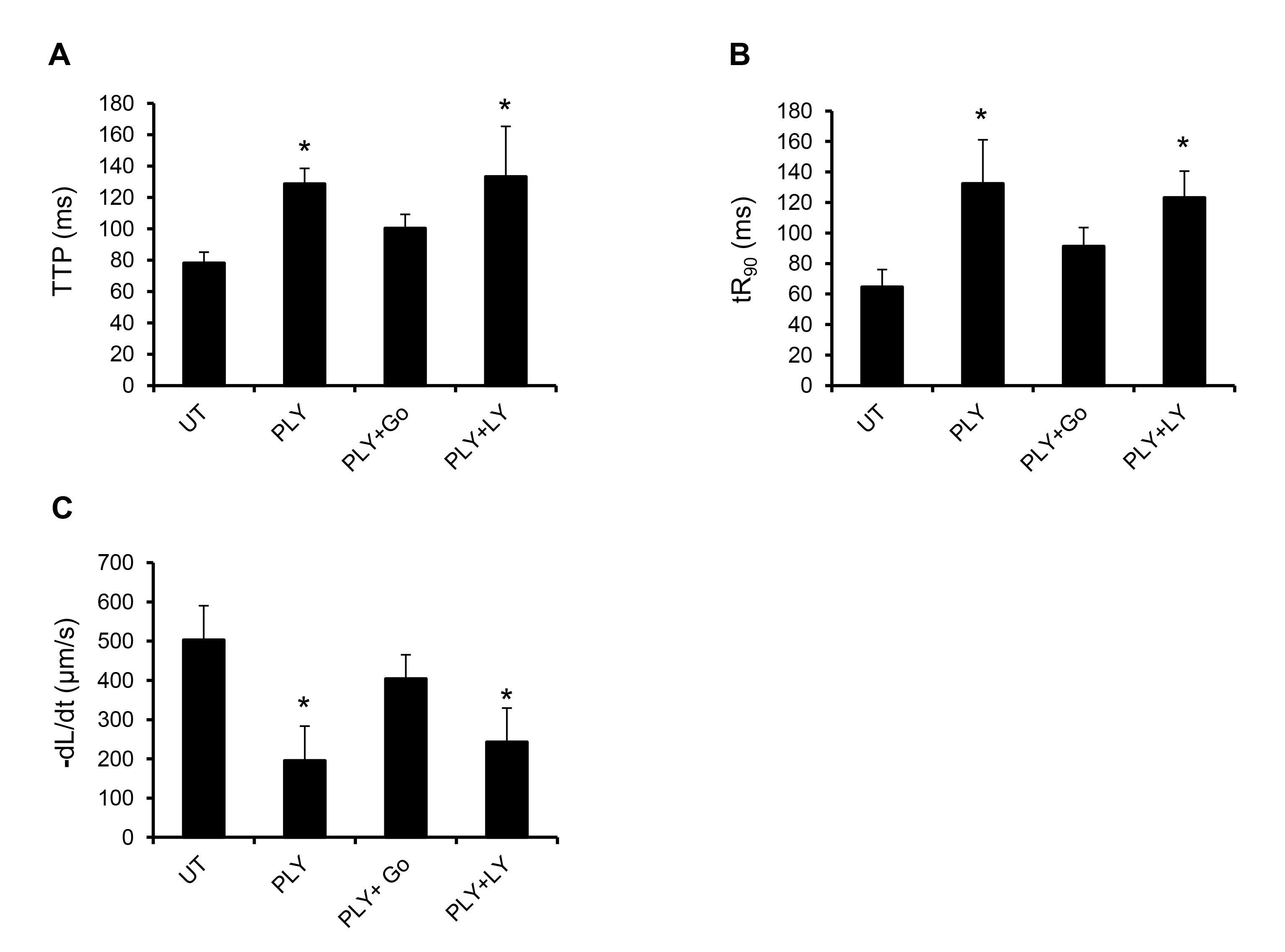

Supplement: S4 Fig — Effects of PLY (1 μg/ml) ± Go6976 (5 nM) or LY333531 (10 nM) on time to peak (TTP) (A), time to 90% re-lengthening (tR90) (B) and the maximum velocity of re-lengthening (-dL/dt) (C) of HL-1 cells after 30 min treatment. Data are presented as Mean±SD. *p<0.05 ANOVA test. (n = 9). (TIF) [file ppat.1004836.s004.tif]
